# Clinical and neurophysiological effects of manual acupuncture for upper-limb motor dysfunction after ischemic stroke: a three-arm randomized controlled trial

**DOI:** 10.3389/fneur.2026.1886740

**Published:** 2026-08-25

**Authors:** Yu Min, Zhendong Huang, Ju Sun, Zetong Peng, Hongli Guan, Yuying Gong, Kaifeng Guo

**Affiliations:** Department of Rehabilitation Medicine, The Affiliated Panyu Central Hospital, Guangzhou Medical University, Guangzhou, Guangdong, China

**Keywords:** functional near-infrared spectroscopy, ischemic stroke, manual acupuncture, neuroplasticity, surface electromyography, upper-limb motor dysfunction

## Abstract

**Background:**

Upper-limb motor dysfunction is common after ischemic stroke. Evidence separating acupuncture effects from nonspecific needling effects remains limited. This study evaluated manual acupuncture combined with conventional rehabilitation in patients with poststroke upper-limb motor dysfunction.

**Methods:**

This single-center, assessor-blinded, three-arm randomized trial enrolled and randomized 84 patients with unilateral upper-limb impairment within 3 months after ischemic stroke. Participants were assigned 1:1:1 to conventional rehabilitation alone, sham acupuncture plus conventional rehabilitation, or manual acupuncture plus conventional rehabilitation for 4 weeks. All randomized participants were included in the full analysis set. Manual acupuncture was applied at GV20, GV14, LI11, PC6, ST36, and SP6 with manipulation to elicit de qi. Sham acupuncture used superficial nonacupoint needling without manipulation. The primary outcome was change in Fugl-Meyer Assessment of the Upper Extremity (FMA-UE) score. Secondary outcomes included Modified Barthel Index (MBI), absolute surface electromyography (sEMG) RMS amplitudes during passive stretch and voluntary contraction, functional near-infrared spectroscopy-measured cortical activation and resting-state functional connectivity, and adverse events.

**Results:**

Manual acupuncture produced greater FMA-UE improvement than conventional rehabilitation and sham acupuncture, with between-group differences of 9.64 points (95% CI, 5.62 to 13.66; Bonferroni-adjusted *p* < 0.001) and 8.57 points (95% CI, 4.55 to 12.59; Bonferroni-adjusted *p* < 0.001), respectively. MBI scores also improved more in the manual acupuncture group, with differences of 7.32 points versus conventional rehabilitation (95% CI, 1.52 to 13.13; Bonferroni-adjusted *p* = 0.040) and 11.29 points versus sham acupuncture (95% CI, 5.48 to 17.09; Bonferroni-adjusted *p* < 0.001). Manual acupuncture was associated with larger increases in unnormalized biceps brachii and triceps brachii RMS amplitudes during voluntary contraction. Functional near-infrared spectroscopy showed greater ipsilesional prefrontal cortex and primary motor cortex activation after false discovery rate correction, with altered prefrontal and sensorimotor connectivity. Sham acupuncture did not differ significantly from conventional rehabilitation for most outcomes. Three mild cases of subcutaneous bleeding occurred, with no serious adverse events.

**Conclusion:**

Manual acupuncture combined with conventional rehabilitation improved upper-limb motor function and activities of daily living after ischemic stroke. These effects were accompanied by larger increases in proximal-muscle RMS amplitudes during voluntary contraction and by fNIRS-based changes in cortical activation and functional connectivity.

**Clinical trial registration:**

https://itmctr.ccebtcm.org.cn/, Identifier, ITMCTR2025002121.

## 1 Introduction

Stroke remains a major cause of mortality and long-term disability worldwide. The Global Burden of Disease 2021 analysis estimated 93.8 million prevalent stroke cases and 11.9 million incident stroke events globally in 2021, with ischemic stroke accounting for the largest proportion of new stroke cases (1). In China, stroke has also represented one of the leading causes of death and disability among adults since 2015, making post-stroke rehabilitation a continuing public health priority (2). Although advances in acute reperfusion therapy, secondary prevention, and stroke-unit care have improved survival, many survivors continue to live with persistent neurological deficits (3). Among these deficits, upper-limb motor dysfunction is particularly disabling because it directly affects reaching, grasping, object manipulation, dressing, eating, washing, and other activities of daily living (4).

Upper-limb impairment is one of the most common and challenging consequences of ischemic stroke. Previous rehabilitation studies have reported that upper-limb motor impairment occurs in approximately 50–80% of patients during the acute stage and may persist in 40–50% of patients during the chronic stage (5). Compared with lower-limb recovery, restoration of upper-limb function is often slower and less complete, especially for selective movement and hand function (6). The difficulty of upper-limb recovery is closely related to corticospinal tract injury, impaired voluntary motor control, abnormal muscle tone, reduced sensorimotor integration, and maladaptive reorganization of bilateral motor networks. Neuroimaging studies have shown that post-stroke motor recovery involves dynamic reorganization of ipsilesional and contralesional motor networks, while interhemispheric imbalance may contribute to inefficient or compensatory motor control (7). Therefore, improving upper-limb motor function remains a central but unresolved goal of stroke rehabilitation.

Conventional rehabilitation is the cornerstone of post-stroke motor recovery. Physical therapy, occupational therapy, task-oriented training, repetitive motor practice, neuromuscular facilitation, constraint-induced movement therapy, robotic-assisted training, and other structured interventions are widely used to improve motor performance and activities of daily living. Clinical guidelines emphasize early, coordinated, and goal-oriented rehabilitation after stroke (8). However, the therapeutic response to conventional rehabilitation is heterogeneous. Some patients enter a functional plateau despite regular training, particularly when severe paresis, spasticity, fatigue, poor motor intention, or insufficient treatment intensity limits active participation (3, 9). These limitations have encouraged the development of adjunctive therapies that can be combined with conventional rehabilitation to enhance neuroplasticity, improve neuromuscular activation, and increase functional recovery.

Acupuncture has been increasingly investigated as an adjunctive intervention for post-stroke motor rehabilitation. From a biomedical perspective, acupuncture can be understood as a patterned peripheral somatosensory stimulation that may influence spinal excitability, corticospinal output, cerebral hemodynamics, and sensorimotor network reorganization (10, 11). Recent systematic reviews suggest that acupuncture combined with rehabilitation may improve neurological function and limb motor outcomes after ischemic stroke, but the overall certainty of evidence remains limited by small sample sizes, heterogeneous acupuncture prescriptions, difficulty in blinding, variable sham controls, and frequent reliance on clinical scales alone (12, 13). Therefore, well-designed controlled trials using standardized acupuncture procedures, appropriate comparators, and objective neurophysiological outcomes are still needed.

The acupuncture protocol used in the present study is derived from the clinical approach traditionally known as Tongdu Tiaoshen acupuncture. It can be described as a standardized manual acupuncture protocol combining cranial-cervical and limb acupoints to provide repeated somatosensory input related to upper-limb motor control, and systemic rehabilitation support. The core acupoints include Baihui (GV20), Dazhui (GV14), Quchi (LI11), Neiguan (PC6), Zusanli (ST36), and Sanyinjiao (SP6). GV20 is located at the vertex and GV14 at the posterior midline below the seventh cervical spinous process; both are commonly used in acupuncture studies related to cerebral ischemia and central nervous system regulation. Experimental studies have reported that stimulation at GV20 and GV14 may improve cerebral perfusion, reduce ischemic injury, and exert neuroprotective effects in animal models of cerebral ischemia–reperfusion injury (14). LI11 and ST36 are among the most frequently studied acupoints in experimental ischemic stroke research, and electroacupuncture at these points has been associated with reactive astrocyte proliferation, brain-derived neurotrophic factor expression, and neuroprotection after ischemia–reperfusion injury (15). PC6 has also been studied in experimental stroke models, with reported effects on neurological outcomes, infarct volume, inflammatory responses, and oxidative stress (16). However, much of the existing mechanistic evidence is derived from animal experiments, electroacupuncture protocols, or isolated acupoint combinations. Whether a complete standardized manual acupuncture protocol can improve upper-limb recovery after ischemic stroke, beyond nonspecific shallow-needling effects and conventional rehabilitation alone, remains insufficiently clarified.

In addition to clinical outcomes, objective measures are needed to clarify how acupuncture may influence post-stroke motor recovery. Surface electromyography (sEMG) provides a non-invasive method for quantifying muscle electrical activity during passive stretch and voluntary contraction, thereby reflecting abnormal muscle tone, motor-unit recruitment, and neuromuscular control. Recent acupuncture trial protocols for post-stroke upper-limb dysfunction have emphasized the value of combining validated clinical scales with sEMG to improve the objectivity of efficacy assessment (17). Functional near-infrared spectroscopy (fNIRS) is another suitable tool for stroke rehabilitation research because it can noninvasively monitor task-related cortical hemodynamic responses and resting-state functional connectivity (FC) with relatively good tolerance in patients with motor impairment (18). A recent fNIRS study reported that auricular acupuncture improved upper-limb motor deficits after stroke and was associated with increased activation of the primary motor cortex, suggesting that M1 may be an important cortical node in acupuncture-related motor recovery (19). Moreover, fNIRS can dynamically characterize cortical hemodynamic responses and functional connectivity patterns during post-stroke rehabilitation, thereby providing objective and quantifiable exploratory biomarkers for evaluating rehabilitation-related changes and generating mechanistic hypotheses (20).

Based on these considerations, the present three-arm clinical study was designed to evaluate the effects of a standardized acupuncture protocol combined with conventional rehabilitation in patients with upper-limb motor dysfunction after ischemic stroke. Participants were randomly allocated to one of three groups: an acupuncture group, a sham acupuncture group receiving superficial needling at non-acupoints, or a conventional rehabilitation group. This design allowed us to distinguish the specific therapeutic effect of the standardized acupuncture protocol from nonspecific needling-related effects and from the expected effects of conventional rehabilitation alone. Clinical outcomes included the Fugl–Meyer Assessment of the Upper Extremity and the Modified Barthel Index (MBI), while sEMG and fNIRS were used as secondary exploratory measures to characterize peripheral neuromuscular activity, cortical hemodynamic responses, and resting-state functional connectivity. We hypothesized that acupuncture combined with conventional rehabilitation would lead to greater improvements in upper-limb motor function and activities of daily living. We further explored whether these clinical improvements were accompanied by changes in upper-limb muscle activity quantified by sEMG and cortical hemodynamic responses assessed using fNIRS.

## 2 Methods

### 2.1 Participants and trial design

Patients with ischemic stroke and unilateral upper-limb motor impairment were recruited from the Departments of Rehabilitation Medicine and Geriatric Rehabilitation at the Affiliated Panyu Central Hospital, Guangzhou Medical University, Guangzhou, China, between December 2025 and April 2026. This was a single-center, assessor-blinded, three-arm randomized controlled trial. Eligible participants were allocated in a 1:1:1 ratio to acupuncture, sham acupuncture, or conventional rehabilitation. The study was approved by the Ethics Committee of the Affiliated Panyu Central Hospital, Guangzhou Medical University (approval No. PYRC-2023-197). Written informed consent was obtained from all participants or their legal representatives. This trial was registered with the International Traditional Medicine Clinical Trial Registry (ITMCTR; registration number: ITMCTR2025002121; registration date: November 3, 2025; URL: https://itmctr.ccebtcm.org.cn/). The registration was completed before enrollment of the first participant. This trial was reported in accordance with the CONSORT 2025 statement (21). A completed CONSORT checklist is provided as Supplementary Table S1.

Ischemic stroke was diagnosed by neurologists and confirmed by computed tomography or magnetic resonance imaging. Patients were eligible if they were right-handed adults aged 30–85 years, were within 3 months after stroke onset, and clinically stable with clear consciousness and stable vital signs. Eligible patients had post-stroke unilateral upper-limb motor dysfunction, with a Brunnstrom stage of ≥ II and a Manual Muscle Testing (MMT) grade of ≤4 in the affected upper limb. Participants were also required to have a Mini-Mental State Examination (MMSE) score within the normal range and to be able to understand instructions and complete the intervention and assessments. Patients were excluded if they had any of the following conditions: pre-existing upper-limb dysfunction unrelated to the index stroke; concomitant cerebral hemorrhage or hemorrhagic transformation of the index ischemic stroke on computed tomography or magnetic resonance imaging; previous traumatic brain injury or other central nervous system disease; severe systemic disease, active infection, malignancy, or coagulation disorder; skin infection, ulceration, or other conditions unsuitable for needling at the planned acupuncture sites; or a known history of needle-related syncope.

### 2.2 Sample size calculation

The sample size was calculated for the primary outcome, the change in FMA-UE score from baseline to week 4. The primary comparison for sample size estimation was manual acupuncture plus conventional rehabilitation versus sham acupuncture plus conventional rehabilitation. Based on previous literature (22), the expected between-group difference was 6.9 points and the estimated common standard deviation was 8.5 points, corresponding to a standardized effect size of approximately 0.81. With a two-sided alpha level of 0.05, 80% statistical power, and a 1:1:1 allocation ratio, 25 participants per group were required for the primary comparison. Allowing for an anticipated dropout rate of 10%, the final target sample size was set at 28 participants per group, for a total of 84 participants.

### 2.3 Randomization and masking

The random allocation sequence was generated by an independent researcher who was not involved in recruitment, treatment, outcome assessment, or data analysis, using a computer-generated random number sequence. Participants were randomly assigned to the three groups in a 1:1:1 ratio using permuted block randomization with a fixed block size of 6, without stratification. The allocation sequence was kept by the independent researcher and was inaccessible to personnel involved in enrollment and assessment. Group assignments were placed in sequentially numbered, opaque, sealed envelopes. After eligibility confirmation and baseline assessment, the study coordinator opened the next envelope in sequence and assigned the participant to the corresponding group.

Because of the nature of the intervention, acupuncturists could not be masked. Participants in the acupuncture and sham acupuncture groups were informed that they would receive one of two acupuncture-like interventions, but they were not told which was expected to be therapeutically active. The two needling interventions were performed in separate rooms or at different time periods, and participants were asked not to discuss treatment details with assessors. After completion of the intervention and before disclosure of allocation, participants in the acupuncture and sham acupuncture groups were asked to guess whether they had received manual acupuncture, sham acupuncture, or were uncertain. Participants in the conventional rehabilitation group could not be masked because they did not receive a needling procedure. Outcome assessors, sEMG and fNIRS operators, data managers, and statisticians were masked to group allocation.

### 2.4 Interventions

All participants received routine medical management for ischemic stroke and a standardized conventional rehabilitation program. The program consisted of 20 sessions over 4 weeks, 5 days per week, approximately 60 min per session. Conventional rehabilitation was delivered by licensed rehabilitation therapists who had received standardized stroke rehabilitation training and trial specific protocol training. The same rehabilitation protocol, treatment dose, and progression rules were applied across the three groups, and therapists were instructed not to modify rehabilitation content according to group allocation. Each session included affected upper limb positioning, range of motion training, task oriented upper limb practice, and progressive strengthening or endurance exercises within each participant’s tolerated range. Training intensity was adjusted to elicit active effort without excessive fatigue, unstable vital signs, or a marked increase in abnormal muscle tone. Task difficulty was progressed when participants showed good task completion across two consecutive sessions, with stable vital signs, acceptable fatigue, and no unsafe compensatory movement. Progression involved reducing assistance or increasing range of motion, repetitions, resistance, or related task demand. Session duration, completed components, progression, and adverse symptoms were recorded in standardized treatment logs. All needling procedures were performed by licensed acupuncturists with trial-specific training.

In the acupuncture group, sterile disposable stainless-steel needles were used (Hanyi; Tianjin Huahong Medical Co., Ltd., Tianjin, China; 0.30 × 25 mm or 0.30 × 40 mm). The acupoints were GV20, GV14, LI11, PC6, ST36, and SP6, with locations and insertion depths specified in Table 1. Limb acupoints were needled bilaterally. Manual manipulation was applied after insertion and every 10 min during the 30 min retention period to elicit de qi, defined as soreness, numbness, heaviness, or distension.

**Table 1:** Locations and needling depths of acupoints in the acupuncture group.

Acupoint	International code	Location	Needling depth
Baihui	GV20	On the head, 5 body-cun superior to the anterior hairline, approximately at the midpoint of the line connecting the auricular apices;	0.5–0.8 body-cun
Dazhui	GV14	On the posterior midline, in the depression below the spinous process of the seventh cervical vertebra;	0.5–1.2 body-cun
Quchi	LI11	At the lateral end of the cubital crease, midway between Chize and the lateral epicondyle of the humerus;	0.5–1.2 body-cun
Neiguan	PC6	On the anterior forearm, 2 body-cun proximal to the wrist crease, between the tendons of palmaris longus and flexor carpi radialis;	0.5–0.8 body-cun
Zusanli	ST36	On the anterolateral aspect of the leg, 3 body-cun inferior to Dubi, one finger-breadth lateral to the anterior crest of the tibia;	0.8–1.2 body-cun
Sanyinjiao	SP6	On the medial aspect of the leg, 3 body-cun proximal to the prominence of the medial malleolus, posterior to the medial border of the tibia;	0.5–1.2 body-cun

In the sham acupuncture group, sterile disposable stainless-steel needles were inserted superficially at six nonacupoint sites near but distinct from the corresponding acupoints (Hanyi; Tianjin Huahong Medical Co., Ltd., Tianjin, China; 0.25 × 13 mm). Sham site locations and insertion depth are shown in Table 2. No manual manipulation was applied, and no attempt was made to elicit de qi. Retention time, treatment schedule, setting, and practitioner–participant contact were matched to those in the acupuncture group; needle handles were lightly touched every 10 min during retention to mimic procedural contact.

**Table 2:** Locations and needling depths of nonacupoint sites in the sham acupuncture group.

Sham site	Location	Depth
Sham 1	On the head, 2 body-cun lateral to GV20	0.2 body-cun
Sham 2	On the posterior neck, 1 body-cun lateral to GV14	0.2 body-cun
Sham 3	On the upper limb, 1 body-cun inferior to LI11	0.2 body-cun
Sham 4	On the anterior forearm, 0.5 body-cun radial to PC6	0.2 body-cun
Sham 5	On the anterolateral leg, 1 body-cun lateral to ST36	0.2 body-cun
Sham 6	On the medial leg, 0.5 body-cun anterior to SP6	0.2 body-cun

### 2.5 Outcomes

Assessments were performed before randomization and after 4 weeks of intervention. The primary outcome was the change in FMA-UE score from baseline to week 4. Secondary outcomes included the MBI, sEMG-derived root mean square values of selected upper-limb muscles during passive stretch and active contraction, task-related cortical activation measured by fNIRS, resting-state FC, and adverse events. All outcomes followed the same assessment schedule; if a participant was discharged before the scheduled week-4 assessment, endpoint assessments were performed immediately before discharge, and the actual assessment timing was recorded.

### 2.6 sEMG acquisition and analysis

sEMG was recorded from the affected-side biceps brachii, triceps brachii, wrist flexors, and wrist extensors using a multichannel sEMG system (Thought Technology, Canada). Recordings were performed in a quiet room at 22–25 °C and 40–60% relative humidity. After skin preparation, disposable Ag/AgCl bipolar electrodes were placed over the muscle belly parallel to the muscle fibers, with an inter-electrode distance of approximately 2 cm.

After a 20-s resting baseline, participants were instructed to relax the affected limb during passive testing. The examiner moved the target joint smoothly through the participant’s available range over 5 s: passive elbow extension for the biceps brachii, passive elbow flexion for the triceps brachii, passive wrist extension for the wrist flexors, and passive wrist flexion for the wrist extensors. During active testing, participants performed resisted elbow flexion, elbow extension, wrist flexion, and wrist extension, respectively, with maximal effort within their available range. Each task was repeated three times.

Raw sEMG signals were sampled at 2048 Hz, band-pass filtered at 20–450 Hz, and notch filtered at 50 Hz. Signals were full-wave rectified, and RMS amplitude was calculated using a 250-ms moving window with 50% overlap. The mean RMS amplitude across the three trials was analyzed in μV. The amplitudes were not normalized to maximum voluntary contraction (MVC).

### 2.7 fNIRS acquisition

**Figure 1:**
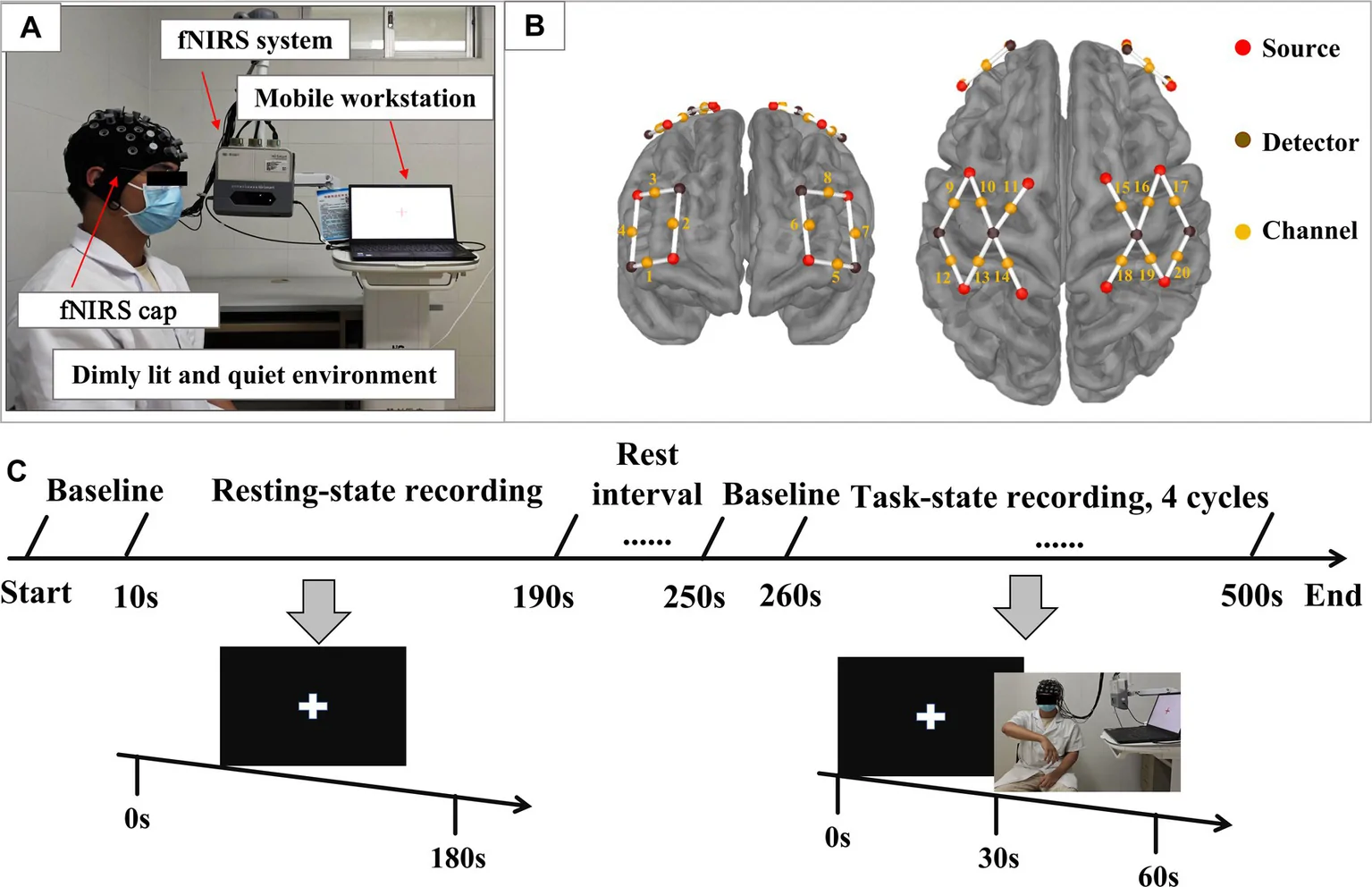
fNIRS acquisition setup, probe montage, and experimental procedure. **(A)** fNIRS recording setup in a dimly lit and quiet environment. **(B)** Probe montage and measurement-channel locations. Red dots indicate sources, brown dots indicate detectors, yellow dots indicate measurement channels, and numbers indicate channel numbers. **(C)** Experimental procedure, including a 10 s baseline, 180 s resting-state recording, a 60 s rest interval, a 10 s task baseline, and four task-state cycles. Each cycle consisted of a 30 s rest block followed by a 30 s block of the composite affected upper-limb movement task.

Cortical hemodynamic activity was recorded using a continuous-wave fNIRS system (NirSmart-4000B, Danyang Huichuang Medical Equipment Co., Ltd., China). The system used two wavelengths, 730 and 850 nm, with a sampling rate of approximately 11 Hz. Twelve sources and eight detectors were arranged to form 20 measurement channels, with a source-detector distance of approximately 3 cm. A customized head cap was positioned according to the international 10–20 system, with Cz as the reference point. The probe layout covered the bilateral prefrontal cortex, premotor cortex, primary motor cortex, and primary somatosensory cortex. The fNIRS acquisition setup and probe montage are shown in Figures 1A,B, and the correspondence among channels, regions of interest, and Brodmann areas is presented in Table 3. The head cap was covered with a black cloth during recording to reduce ambient light interference.

**Table 3:** Correspondence among fNIRS channels, ROIs, and Brodmann areas.

ROI	Channel	BA	ROI	Channel	BA
Left hemisphere			Right hemisphere		
PFC	5	BA10	PFC	1	BA10
	6	BA10		2	BA10
	7	BA46, BA10		3	BA46, BA10
	8	BA10		4	BA10
PM	15	BA6	PM	9	BA6
	16	BA6		10	BA6
	17	BA6		11	BA6
M1	18	BA4	M1	13	BA4
	19	BA4		14	BA4
S1	20	BA1, BA2, BA3	S1	12	BA1, BA2, BA3, BA4

ROI, region of interest; BA, Brodmann area; PFC, prefrontal cortex; PM, premotor cortex; M1, primary motor cortex; S1, primary somatosensory cortex.

Both resting-state and task-state fNIRS data were acquired. During resting-state recording, participants sat or lay quietly for 3 min, remained awake and relaxed, and avoided speaking or moving. During task-state recording, a single composite affected upper-limb movement task incorporating shoulder abduction, shoulder flexion, and elbow flexion was assessed using a block design. A total of 4 cycles were performed, with each cycle consisting of a 30-s rest block followed by a 30-s movement block. Participants with limited active movement were instructed to attempt the movement as fully as possible within their available range while keeping the unaffected limb relaxed. The overall experimental procedure is illustrated in Figure 1C.

### 2.8 fNIRS preprocessing and analysis

fNIRS data were processed using NirSpark software(NirSmart-4000B, Danyang Huichuang Medical Equipment Co., Ltd., China). Channels with absent signals, obvious noise, or a coefficient of variation greater than 20% were excluded. Raw light intensity signals were converted to optical density. Motion artifacts were corrected using a moving standard deviation and spline interpolation method, with a standard deviation threshold of 6 and an amplitude threshold of 0.5. Signals were then band-pass filtered at 0.01–0.2 Hz. Optical density data were converted to relative changes in oxygenated hemoglobin and deoxygenated hemoglobin using the modified Beer–Lambert law, with the differential pathlength factor set to 6. Subsequent analyses focused on oxygenated hemoglobin because of its higher sensitivity to task-related cortical hemodynamic changes.

Before group-level analyses, channels and regions of interest (ROIs) were recoded according to lesion side, so that the hemisphere coded as left consistently represented the ipsilesional hemisphere across participants.

For task-related activation, the 2-s period before each movement block was used as baseline. The mean task-related change in oxygenated hemoglobin was calculated for each channel and then averaged within each region of interest. For resting-state FC, Pearson correlation coefficients were calculated between oxygenated hemoglobin time series of channels and regions of interest. Correlation coefficients were transformed to Fisher z values before statistical analysis.

### 2.9 Safety monitoring

Adverse events were monitored throughout the intervention period. Participants were asked about discomfort after each session, and needling sites were inspected regularly. Needle-related adverse events included pain, bleeding, bruising, dizziness, fainting, infection, stuck needle, and broken needle. Serious adverse events, including recurrent stroke or clinically significant deterioration, were reported to the ethics committee according to institutional requirements.

### 2.10 Statistical analysis

All statistical analyses were performed using Python version 3.9.7. Data processing and statistical modeling were conducted using pandas, NumPy, SciPy, and statsmodels. Continuous variables were summarized using the mean and standard deviation (SD) when approximately normally distributed and the median and interquartile range (IQR) when skewed. Categorical variables were summarized as n (%). Continuous outcomes were analyzed using linear mixed-effects models, with the outcome value as the dependent variable, group, time, and group-by-time interaction as fixed effects, and participant as a random intercept. The group-by-time interaction was assessed using Wald tests. When a significant interaction was observed, *post-hoc* pairwise comparisons of changes from baseline were performed, and *p* values were adjusted using the Bonferroni method. For fNIRS activation and FC outcomes, false discovery rate correction was applied to the interaction p values across regions of interest or connectivity pairs using the Benjamini–Hochberg method. Ordinal variables were analyzed using the Kruskal–Wallis test for overall between-group comparisons and the Mann–Whitney U test for pairwise comparisons. Categorical variables were analyzed using the chi-square test or Fisher’s exact test, as appropriate. For participant allocation guesses in the acupuncture and sham acupuncture groups, the association between actual allocation and guessed allocation was assessed using the chi-square test, and blinding success within each needling group was quantified using Bang’s blinding index (23). All tests were two-sided, and *p* < 0.05 was considered statistically significant. The primary analysis was performed in the full analysis set, defined as all randomized participants with observed baseline and endpoint data, analyzed according to their randomized groups.

## 3 Results

### 3.1 Participants and data availability

Between December 2025 and April 2026, 96 patients were assessed for eligibility, and 12 were excluded before randomization because of a history of needle-related syncope (*n* = 2), coagulation disorder (*n* = 5), concomitant cerebral hemorrhage or hemorrhagic transformation (*n* = 4), or a local skin lesion at a planned needling site (*n* = 1). A total of 84 eligible participants were randomized, with 28 assigned to the conventional rehabilitation group, 28 to the sham acupuncture group, and 28 to the acupuncture group. Adherence to the rehabilitation protocol was monitored using treatment logs. All randomized participants completed at least 15 of the planned 20 rehabilitation sessions and were included in the full analysis set according to their randomized groups. In the conventional rehabilitation group, all 28 participants completed the 4-week intervention and endpoint assessments at week 4. In the sham acupuncture group, 27 participants completed the 4-week intervention and endpoint assessments at week 4. The remaining participant was discharged after 3 weeks of treatment and underwent endpoint assessments before discharge. In the acupuncture group, 26 participants completed the 4-week intervention and endpoint assessments at week 4. The remaining 2 participants were discharged early, one on day 22 and one on day 25 of treatment, and underwent endpoint assessments before discharge. No participant was lost to follow-up, excluded from the main analysis, or withdrawn because of adverse events. No outcome imputation was performed. Each of the conventional rehabilitation, sham acupuncture, and acupuncture groups included 28 participants in the outcome analyses.

**Figure 2:**
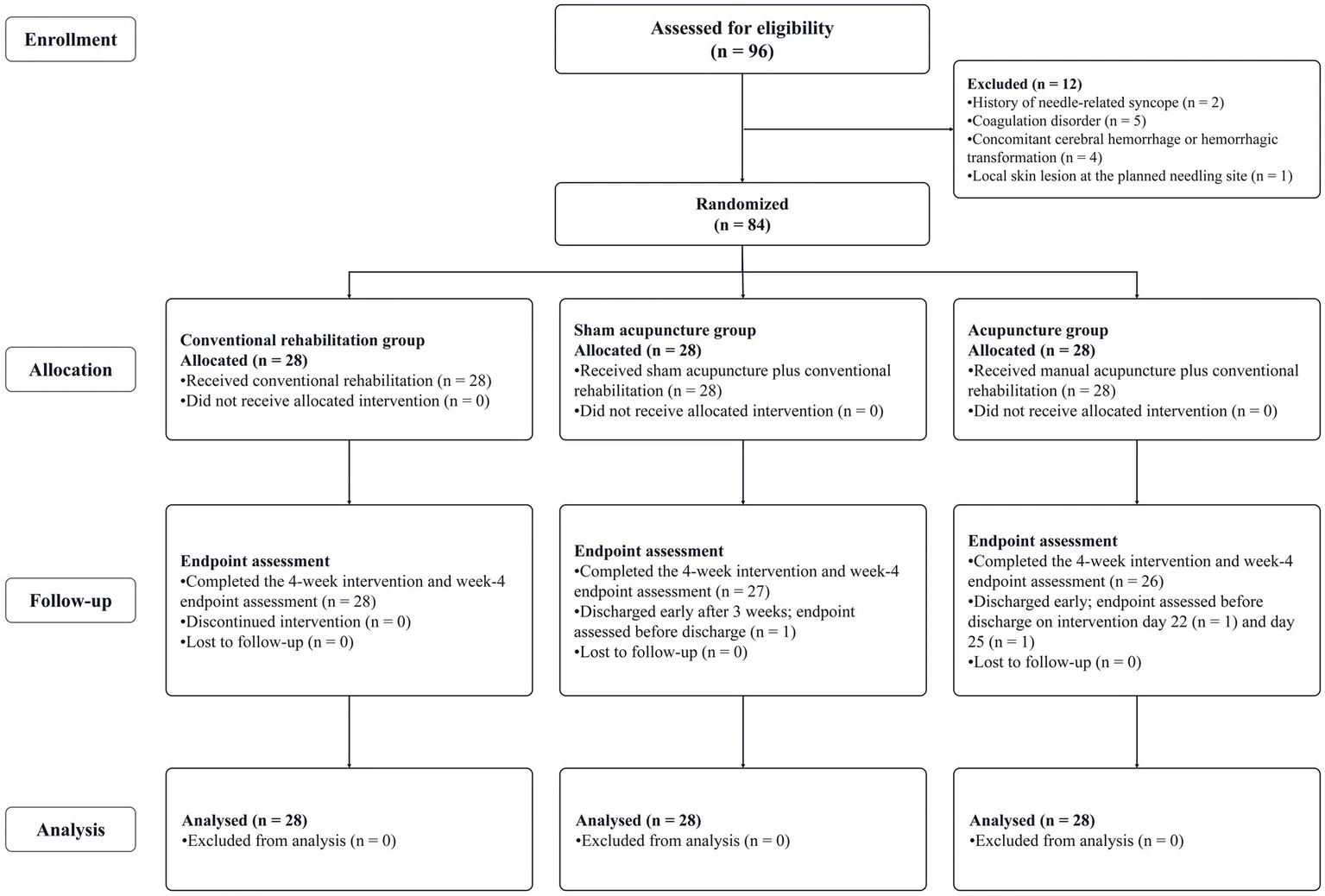
CONSORT flow diagram. All randomized participants completed the final assessment and were included in the analysis.

Baseline demographic characteristics of the participants are summarized in Table 4 and were comparable among the three groups, with no statistically significant differences observed (all *p* > 0.05). Participant allocation guesses were available for all participants in the two needling groups. In the acupuncture group, 14 participants guessed that they had received manual acupuncture, 11 guessed sham acupuncture, and 3 were uncertain. In the sham acupuncture group, 8 participants guessed manual acupuncture, 13 guessed sham acupuncture, and 7 were uncertain. Actual allocation was not significantly associated with guessed allocation (χ^2^ = 3.40, *p* = 0.182). Bang’s blinding index was 0.11 (95% CI, −0.24 to 0.45) in the acupuncture group and 0.18 (95% CI, −0.14 to 0.49) in the sham acupuncture group (Figure 2).

**Table 4:** Baseline demographic and lesion characteristics of participants.

Characteristic	Conventional rehabilitation group	Sham acupuncture group	Acupuncture group	*p*
Age, years	66.18 ± 13.61	64.86 ± 8.35	67.14 ± 9.73	0.730
Male sex, n (%)	17 (60.71)	18 (64.29)	17 (60.71)	0.951
Disease duration, months	2.00 (1.00, 3.00)	2.00 (1.00, 3.00)	2.00 (1.00, 2.75)	0.588
Hemiplegic side				
Left	14 (50.00)	17 (60.71)	12 (42.86)	0.404
Right	14 (50.00)	11 (39.29)	16 (57.14)	
Responsible lesion, n (%)				
Cortical/lobar lesion	5 (17.86)	3 (10.71)	4 (14.29)	0.857
Subcortical white matter lesion	4 (14.29)	4 (14.29)	6 (21.43)	
Deep hemispheric lesion	17 (60.71)	20 (71.43)	15 (53.57)	
Multiple lesions	2 (7.14)	1 (3.57)	3 (10.71)	

Observed values are presented as mean ± SD or median (IQR) for continuous variables, and as n (%) for categorical variables.

### 3.2 Clinical functional outcomes

Clinical function improved after treatment in all three groups, with the largest changes observed in the acupuncture group (Table 5). FMA-UE scores showed a significant group-by-time interaction (Wald χ^2^ = 26.54, *p* < 0.001). Pairwise comparisons of change scores indicated greater improvement in the acupuncture group than in the conventional rehabilitation group (difference in change, 9.64 points; 95% CI, 5.62 to 13.66; Bonferroni-adjusted *p* < 0.001) and the sham acupuncture group (difference in change, 8.57 points; 95% CI, 4.55 to 12.59; Bonferroni-adjusted *p* < 0.001). The sham acupuncture group did not differ significantly from the conventional rehabilitation group (Bonferroni-adjusted *p* > 0.999).

**Table 5:** Clinical functional outcomes at baseline and after the 4-week intervention.

Outcome	Time point	Conventional rehabilitation group	Sham acupuncture group	Acupuncture group	Between-group *p*	Interaction Wald χ^2^	Interaction *p*
FMA-UE	Baseline	22.71 ± 8.10	25.86 ± 10.19	27.07 ± 10.10	0.152	26.54	<0.001
	Post-treatment	31.11 ± 8.08	35.32 ± 7.78	45.11 ± 8.43	<0.001		
	Change from baseline (95% CI)	8.39(6.39, 10.40)	9.46(6.50, 12.43)	18.04(14.19, 21.88)	<0.001		
MBI	Baseline	37.50 ± 16.18	35.36 ± 11.02	41.39 ± 12.24	0.128	14.95	<0.001
	Post-treatment	53.00 ± 6.04	46.89 ± 9.86	64.21 ± 11.31	<0.001		
	Change from baseline (95% CI)	15.50(10.60, 20.40)	11.54(8.07, 15.00)	22.82(18.19, 27.45)	<0.001		

Observed values are presented as mean ± SD or median (IQR); estimated changes from baseline are presented with 95% CIs. FMA-UE, Fugl-Meyer assessment of the upper extremity; MBI, modified barthel index; CI, confidence interval; Wald χ^2^, Wald chi-square statistic.

MBI scores showed a similar pattern. The group-by-time interaction was significant (Wald χ^2^ = 14.95, *p* < 0.001), and the increase in the acupuncture group was greater than that in the conventional rehabilitation group (difference in change, 7.32 points; 95% CI, 1.52 to 13.13; Bonferroni-adjusted *p* = 0.040) and the sham acupuncture group (difference in change, 11.29 points; 95% CI, 5.48 to 17.09; Bonferroni-adjusted *p* < 0.001). No significant difference was detected between the sham acupuncture and conventional rehabilitation groups (Bonferroni-adjusted *p* = 0.542).

### 3.3 sEMG outcomes

During passive stretch, significant group-by-time interactions were found for triceps brachii RMS (Wald χ^2^ = 8.33, *p* = 0.016) and wrist flexor RMS (Wald χ^2^ = 19.75, *p* < 0.001), whereas biceps brachii RMS and wrist extensor RMS did not show significant interactions (Table 6; Supplementary Table S2). For triceps brachii RMS, the change in the conventional rehabilitation group exceeded that in the acupuncture group (difference in change, 9.47 μV; 95% CI, 3.00 to 15.93; Bonferroni-adjusted *p* = 0.012). For wrist flexor RMS, the acupuncture group showed greater change than the conventional rehabilitation group (difference in change, 9.81 μV; 95% CI, 5.31 to 14.32; Bonferroni-adjusted *p* < 0.001) and the sham acupuncture group (difference in change, 7.35 μV; 95% CI, 2.84 to 11.85; Bonferroni-adjusted *p* = 0.004). No significant pairwise difference was observed between sham acupuncture and conventional rehabilitation for either passive-stretch outcome with a significant interaction.

**Table 6:** sEMG change scores during passive stretch.

Outcome	CR change (95% CI)	SA change (95% CI)	MA change (95% CI)	Interaction Wald χ^2^	Interaction *p*
Biceps brachii RMS, μV	3.54(0.44, 6.64)	0.38(−3.67, 4.42)	−0.17(−6.85, 6.51)	1.49	0.475
Triceps brachii RMS, μV	12.17(6.68, 17.66)	8.32(3.94, 12.70)	2.70(−1.98, 7.39)	8.33	0.016
Wrist flexor RMS, μV	−7.44(−11.10, −3.79)	−4.98(−7.91, −2.05)	2.37(−1.18, 5.93)	19.75	<0.001
Wrist extensor RMS, μV	3.40(0.76, 6.03)	1.99(−0.31, 4.29)	0.33(−3.27, 3.93)	2.45	0.294

Estimated changes from baseline are presented with 95% CIs. CR, conventional rehabilitation; SA, sham acupuncture; MA, manual acupuncture; sEMG, surface electromyography; CI, confidence interval. Complete observed baseline, endpoint, and change-score results are provided in Supplementary Table S2.

**Figure 3:**
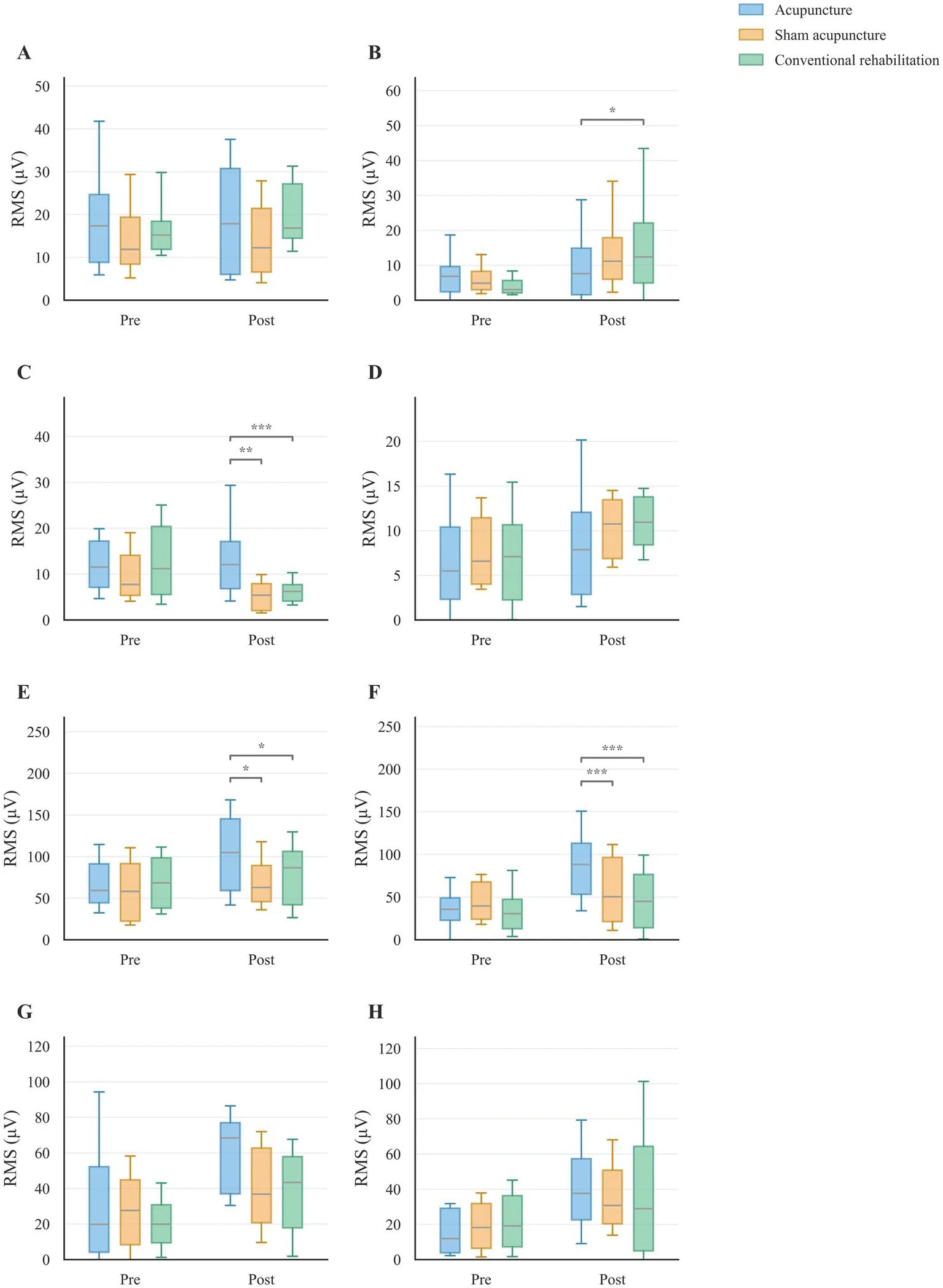
sEMG-derived RMS amplitudes before and after treatment. **(A)** Biceps stretch; **(B)** Triceps stretch; **(C)** Wrist flexor stretch; **(D)** Wrist extensor stretch; **(E)** Biceps contraction; **(F)** Triceps contraction; **(G)** Wrist flexor contraction; **(H)** Wrist extensor contraction. *p < 0.05, **p < 0.01, ***p < 0.001.

During voluntary contraction, significant group-by-time interactions were observed for biceps brachii RMS (Wald χ^2^ = 10.33, *p* = 0.006) and triceps brachii RMS (Wald χ^2^ = 29.94, *p* < 0.001), but not for wrist flexor RMS or wrist extensor RMS (Table 7; Supplementary Table S3). Relative to conventional rehabilitation and sham acupuncture, respectively, the acupuncture group showed larger increases for biceps brachii RMS (differences in change, 30.72 and 28.58 μV; 95% CI, 9.80 to 51.64 and 7.66 to 49.50; Bonferroni-adjusted *p* = 0.012 and *p* = 0.022) and triceps brachii RMS (differences in change, 49.33 and 48.40 μV; 95% CI, 29.12 to 69.54 and 28.19 to 68.61; both Bonferroni-adjusted *p* < 0.001). Sham acupuncture and conventional rehabilitation did not differ significantly for either active-contraction RMS outcome with a significant interaction (Figure 3).

**Table 7:** sEMG change scores during voluntary contraction.

Outcome	CR change (95% CI)	SA change (95% CI)	MA change (95% CI)	Interaction Wald χ^2^	Interaction *p*
Biceps brachii RMS, μV	8.37(−7.40, 24.13)	10.51(−4.55, 25.57)	39.09(22.63, 55.55)	10.33	0.006
Triceps brachii RMS, μV	8.31(−4.80, 21.42)	9.24(−3.14, 21.62)	57.64(38.37, 76.91)	29.94	<0.001
Wrist flexor RMS, μV	15.69(7.67, 23.71)	9.65(0.04, 19.26)	25.17(12.16, 38.19)	4.92	0.086
Wrist extensor RMS, μV	20.02(5.43, 34.60)	17.77(10.37, 25.17)	22.20(15.04, 29.36)	0.40	0.817

### 3.4 Task-related cortical activation assessed by fNIRS

As a secondary exploratory neurophysiological outcome, task-related cortical activation was analyzed using HbO changes within predefined regions of interest. After lesion-side recoding, ipsilesional and contralesional regions were described consistently across participants. After false discovery rate correction, significant group-by-time interactions were retained only for ipsilesional prefrontal cortex (PFC; Wald χ^2^ = 40.56, *p* < 0.001, FDR-adjusted *p* < 0.001) and ipsilesional primary motor cortex (M1; Wald χ^2^ = 9.07, *p* = 0.011, FDR-adjusted *p* = 0.043; Table 8; Supplementary Table S4).

**Table 8:** Task-related fNIRS cortical activation by ROI, ΔHbO (10^−2^ mmol/L·mm).

ROI	CR change (95% CI)	SA change (95% CI)	MA change (95% CI)	Interaction Wald χ^2^	Interaction *p*	FDR-adjusted *p*
l-PFC	−1.22(−3.05, 0.60)	−0.63(−2.61, 1.35)	6.58(4.36, 8.80)	40.56	<0.001	<0.001
r-PFC	−1.56(−4.77, 1.66)	1.05(−1.94, 4.04)	−0.85(−3.18, 1.47)	1.93	0.380	0.622
l-M1	1.31(−0.02, 2.64)	1.62(−0.66, 3.89)	4.93(2.76, 7.09)	9.07	0.011	0.043
r-M1	1.20(−0.36, 2.75)	−0.21(−2.34, 1.93)	−1.17(−4.03, 1.70)	2.44	0.296	0.622
l-PM	0.57(−1.61, 2.75)	2.04(0.60, 3.48)	1.01(−1.29, 3.32)	1.23	0.541	0.622
r-PM	−0.50(−1.50, 0.51)	0.26(−1.19, 1.71)	−1.00(−2.88, 0.88)	1.59	0.451	0.622
l-S1	2.68(−0.77, 6.14)	2.49(0.34, 4.64)	4.30(1.04, 7.56)	0.95	0.622	0.622
r-S1	−1.80(−4.78, 1.18)	0.40(−4.06, 4.86)	0.52(−3.35, 4.40)	1.02	0.601	0.622

Estimated changes from baseline are presented with 95% CIs. fNIRS, functional near-infrared spectroscopy; ROI, regions of interest; PFC, prefrontal cortex; M1, primary motor cortex; PM, premotor cortex; S1, primary somatosensory cortex; FDR, false discovery rate; CI, confidence interval; l−/r-, ipsilesional/contralesional after lesion-side recoding. Complete observed baseline, endpoint, and change-score results are provided in Supplementary Table S4.

**Figure 4:**
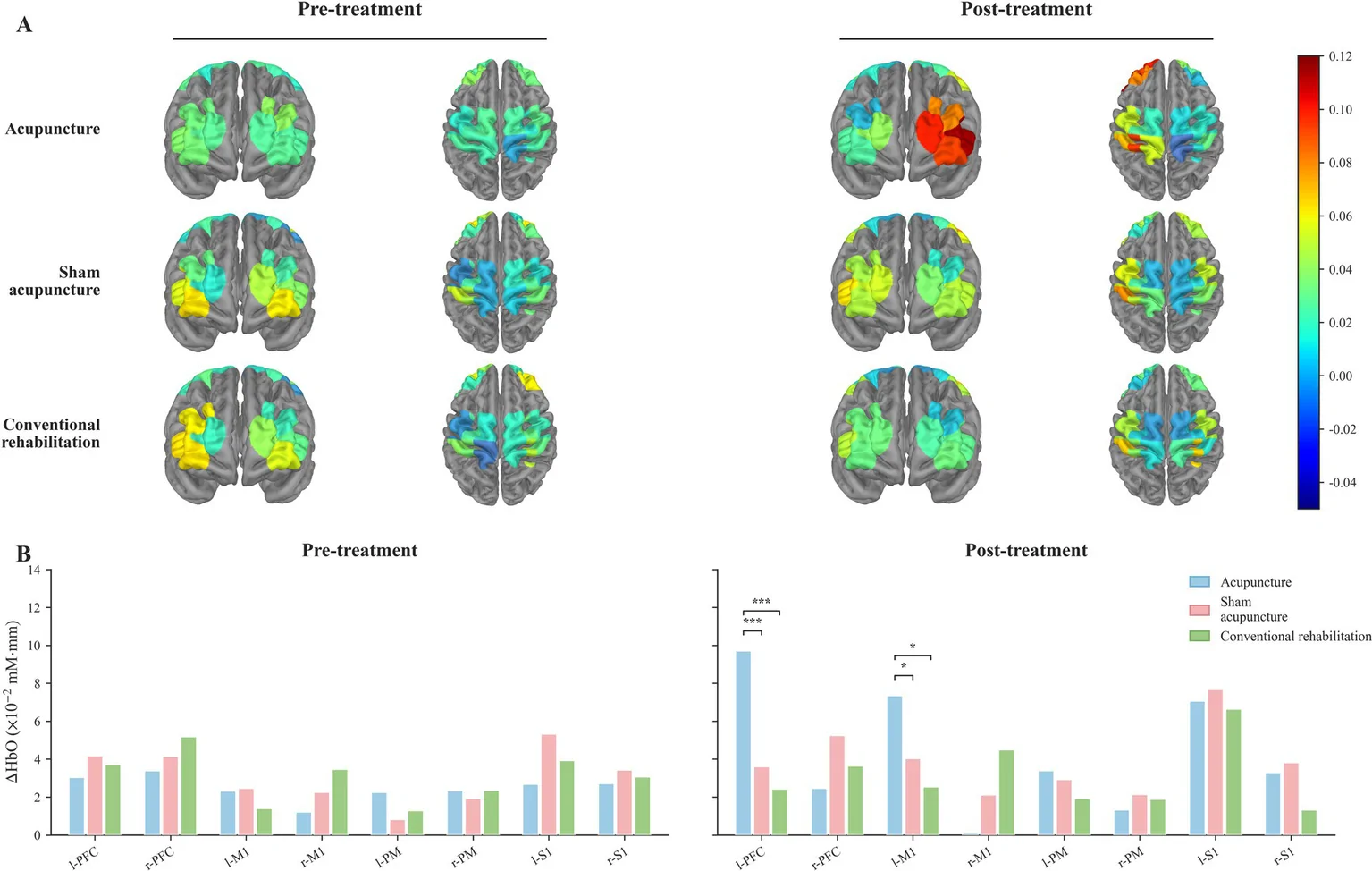
Cortical activation before and after treatment in the three groups. **(A)** Three-dimensional cortical activation maps for the acupuncture, sham acupuncture, and conventional rehabilitation groups before and after treatment. The color scale indicates activation intensity. **(B)** ROI-level ΔHbO values before and after treatment across the three groups. PFC, prefrontal cortex; M1, primary motor cortex; PM, premotor cortex; S1, primary somatosensory cortex; l−/r-, ipsilesional/contralesional after lesion-side recoding. *p < 0.05, **p < 0.01, ***p < 0.001.

For ipsilesional PFC activation, the acupuncture group showed a greater increase than the conventional rehabilitation group (difference in change, 7.80 × 10^−2^ mmol/L·mm; 95% CI, 5.13 to 10.48; Bonferroni-adjusted *p* < 0.001) and the sham acupuncture group (difference in change, 7.21 × 10^−2^ mmol/L·mm; 95% CI, 4.54 to 9.89; Bonferroni-adjusted *p* < 0.001). Ipsilesional M1 activation also increased more in the acupuncture group than in the conventional rehabilitation group (difference in change, 3.62 × 10^−2^ mmol/L·mm; 95% CI, 1.01 to 6.23; Bonferroni-adjusted *p* = 0.020) and the sham acupuncture group (difference in change, 3.31 × 10^−2^ mmol/L·mm; 95% CI, 0.70 to 5.92; Bonferroni-adjusted *p* = 0.039). Sham acupuncture did not differ significantly from conventional rehabilitation for either region. No other task-related ROI showed a significant group-by-time interaction after FDR correction (Figure 4).

### 3.5 Resting-state functional connectivity assessed by fNIRS

In exploratory resting-state FC analyses, four ROI pairs showed significant group-by-time interactions after FDR correction: ipsilesional PFC–contralesional PFC (Wald χ^2^ = 37.67, *p* < 0.001, FDR-adjusted *p* < 0.001), ipsilesional PFC–contralesional premotor cortex (PM; Wald χ^2^ = 20.59, *p* < 0.001, FDR-adjusted *p* < 0.001), contralesional PFC–contralesional PM (Wald χ^2^ = 23.93, *p* < 0.001, FDR-adjusted *p* < 0.001), and ipsilesional M1–ipsilesional primary somatosensory cortex (S1; Wald χ^2^ = 12.13, *p* = 0.002, FDR-adjusted *p* = 0.016; Table 9; Supplementary Table S5).

**Table 9:** Resting-state fNIRS connectivity pairs with FDR-significant group-by-time interactions, Fisher z (×10^−2^).

ROI pair	CR change (95% CI)	SA change (95% CI)	MA change (95% CI)	Interaction Wald χ^2^	Interaction *p*	FDR-adjusted *p*
l-PFC-r-PFC	33.59(14.73, 52.45)	21.09(5.13, 37.05)	−41.49(−64.19, −18.80)	37.67	<0.001	<0.001
l-PFC-r-PM	40.60(11.61, 69.60)	41.90(23.98, 59.81)	−10.33(−25.03, 4.37)	20.59	<0.001	<0.001
r-PFC-r-PM	30.41(14.11, 46.71)	20.81(7.46, 34.16)	−19.91(−38.27, −1.54)	23.93	<0.001	<0.001
l-M1-l-S1	1.57(−17.55, 20.68)	34.82(5.28, 64.37)	74.14(33.98, 114.30)	12.13	0.002	0.016

Estimated changes from baseline are presented with 95% CIs. fNIRS, functional near-infrared spectroscopy; ROI, regions of interest; PFC, prefrontal cortex; M1, primary motor cortex; PM, premotor cortex; S1, primary somatosensory cortex; FDR, false discovery rate; CI, confidence interval; l−/r-, ipsilesional/contralesional after lesion-side recoding. Complete results for all pairs are provided in Supplementary Table S5.

**Figure 5:**
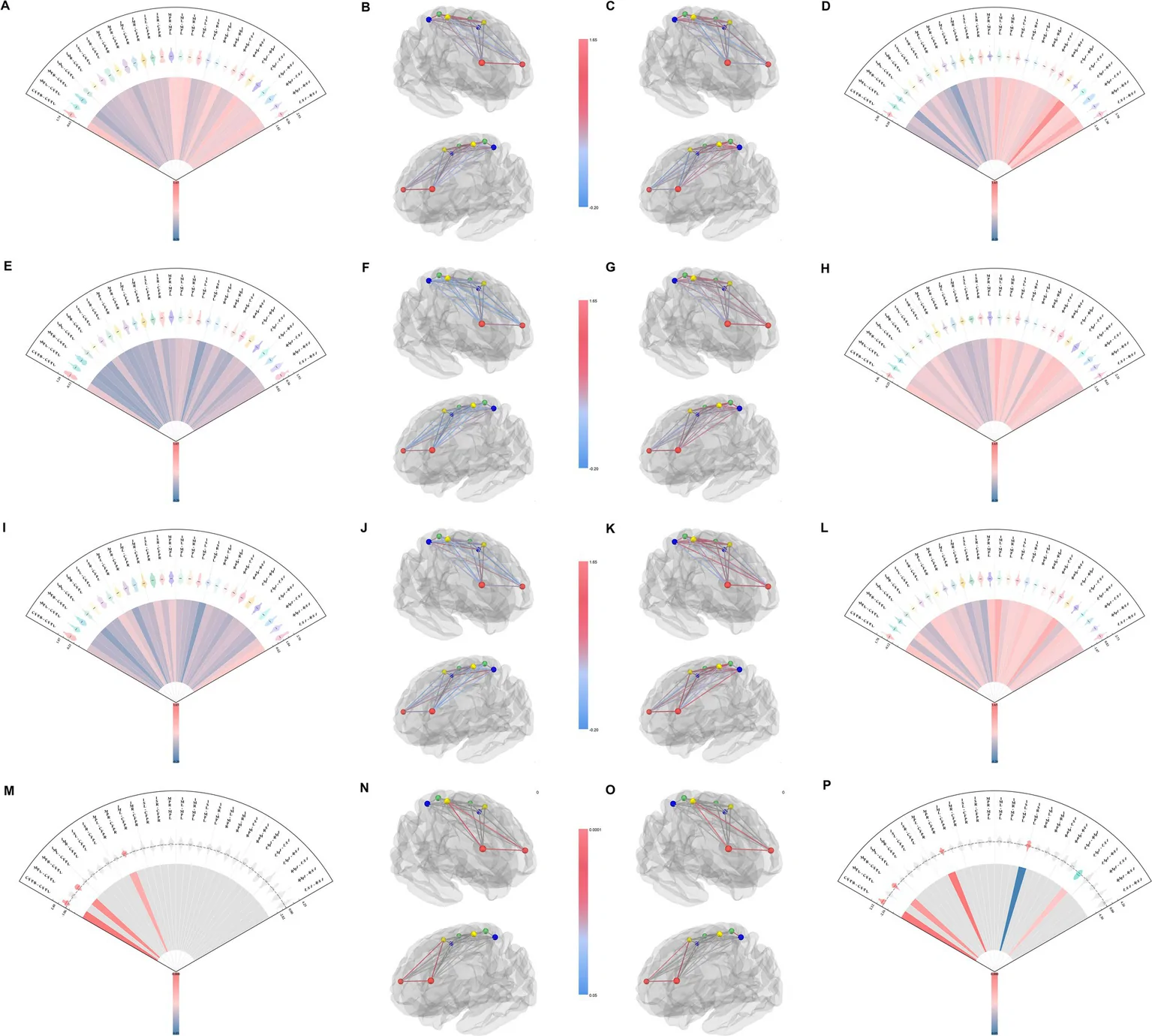
Brain functional connectivity network maps and fan-shaped ROI-to-ROI heatmaps across three treatment groups and two time points. **A–D** show the Acupuncture group, **E–H** show the Sham acupuncture group, and **I–L** show the Conventional rehabilitation group. Within each row, panels are ordered as pre-treatment fan heatmap, pre-treatment brain network map, post-treatment brain network map, and post-treatment fan heatmap. **M** and **N** show the Bonferroni-corrected treatment-effect comparison between Acupuncture and Sham acupuncture. **O** and **P** show the Bonferroni-corrected treatment-effect comparison between Acupuncture and Conventional rehabilitation. PFC, prefrontal cortex; M1, primary motor cortex; PM, premotor cortex; S1, primary somatosensory cortex; l−/r-, ipsilesional/contralesional after lesion-side recoding.

For the PFC–PFC connection and the two PFC–PM connections, the acupuncture group showed significantly smaller connectivity changes than both comparator groups. Specifically, relative to the acupuncture group, the changes in the conventional rehabilitation and sham acupuncture groups, respectively, were greater by 75.09 and 62.58 for ipsilesional PFC–contralesional PFC connectivity (95% CI, 49.39 to 100.78 and 36.89 to 88.28; both Bonferroni-adjusted *p* < 0.001), 50.93 and 52.22 for ipsilesional PFC–contralesional PM connectivity (95% CI, 25.20 to 76.66 and 26.49 to 77.96; Bonferroni-adjusted *p* < 0.001 and *p* < 0.001), and 50.32 and 40.72 for contralesional PFC–contralesional PM connectivity (95% CI, 28.91 to 71.73 and 19.31 to 62.12; both Bonferroni-adjusted *p* < 0.001; Figure 5).

In contrast, ipsilesional M1–ipsilesional S1 connectivity increased more in the acupuncture group than in the conventional rehabilitation group (difference in change, 72.58; 95% CI, 31.68 to 113.47; Bonferroni-adjusted *p* = 0.002). The difference between the acupuncture and sham acupuncture groups did not remain significant after Bonferroni correction (difference in change, 39.32; 95% CI, −1.58 to 80.21; Bonferroni-adjusted *p* = 0.179). No significant differences were found between sham acupuncture and conventional rehabilitation for the four connectivity pairs with significant interactions. Other functional-connectivity outcomes did not show significant group-by-time interactions after FDR correction (Figure 5). Detailed *post-hoc* pairwise comparisons for all outcomes with significant group-by-time interactions are provided in Supplementary Table S6.

### 3.6 Safety outcomes

Minor subcutaneous bleeding at the needling site occurred in two participants in the acupuncture group and one participant in the sham acupuncture group. All events were mild and resolved after routine local management, with no sequelae. No serious adverse events, infections, fainting, stuck needles, broken needles, recurrent stroke, clinically significant deterioration, or withdrawals due to adverse events were reported. Safety outcomes are summarized in Table 10.

**Table 10:** Adverse events during the 4-week intervention.

Adverse event	Conventional rehabilitation group	Sham acupuncture group	Acupuncture group	Overall
Any adverse event, n (%)	0 (0.00)	1 (3.57%)	2 (7.14%)	3 (3.57%)
Subcutaneous bleeding at the needling site, n (%)	0 (0.00)	1 (3.57%)	2 (7.14%)	3 (3.57%)
Serious adverse event, n (%)	0 (0.00)	0 (0.00)	0 (0.00)	0 (0.00)
Withdrawal due to adverse event, n (%)	0 (0.00)	0 (0.00)	0 (0.00)	0 (0.00)

Data are presented as n (%).

## 4 Discussion

### 4.1 Principal findings and clinical relevance

In this assessor-blinded, three-arm randomized controlled trial, the primary finding was that a 4-week standardized manual acupuncture protocol produced greater improvement in FMA-UE scores than sham acupuncture or conventional rehabilitation alone. MBI scores also improved more in the acupuncture group. Secondary exploratory analyses showed that, compared with both comparator groups, the acupuncture group showed larger increases in biceps brachii and triceps brachii RMS amplitudes during voluntary contraction, greater task-related activation of the ipsilesional prefrontal cortex and primary motor cortex, and a more favorable resting-state connectivity pattern involving prefrontal and sensorimotor networks. These sEMG and fNIRS findings accompanied the clinical improvements and should be interpreted as exploratory neurophysiological correlates rather than evidence of an established mechanism. Importantly, sham acupuncture did not differ significantly from conventional rehabilitation for most outcomes, suggesting that the observed benefit was not explained only by additional therapist contact, expectation, or superficial needling. However, this interpretation should be made cautiously because sham credibility was not formally assessed and participants in the conventional rehabilitation group were not blinded.

The clinical effect observed in this trial is relevant for post-stroke upper-limb rehabilitation. Conventional rehabilitation remains the foundation of stroke recovery, and current stroke rehabilitation guidelines emphasize coordinated, task-oriented, and functionally meaningful practice after stroke (24). In the present study, all three groups improved after 4 weeks, which is consistent with the expected effect of structured rehabilitation in patients within the first 3 months after ischemic stroke (25). However, the acupuncture group achieved an FMA-UE change of 18.04 points, compared with 9.46 points in the sham acupuncture group and 8.39 points in the conventional rehabilitation group. The additional improvement over conventional rehabilitation was 9.64 points, and the additional improvement over sham acupuncture was 8.57 points. Previous studies have suggested clinically important differences for the FMA-UE in the range of approximately 5–13 points, depending on stroke stage, severity, and anchor method (26, 27). Therefore, the magnitude of improvement in the acupuncture group is not only statistically significant but also clinically meaningful.

These findings are broadly consistent with previous evidence suggesting that acupuncture can serve as an adjunctive intervention for post-stroke motor rehabilitation. Meta-analyses reported possible beneficial effects of acupuncture in post-stroke rehabilitation, although evidence quality was limited by heterogeneity and methodological weaknesses (28). Recent reviews of post-stroke upper-limb dysfunction suggest that acupuncture, across different modalities, combined with conventional rehabilitation may improve upper-limb motor outcomes, particularly FMA-UE scores, compared with conventional therapy alone, although the lack of protocol standardization remains a major methodological concern (29). The present trial adds to this literature by using a three-arm design with both sham acupuncture and conventional rehabilitation comparators, allowing a clearer distinction between treatment-specific effects, nonspecific needling-related effects, and the expected effect of rehabilitation alone.

The difference between the acupuncture and sham acupuncture groups is particularly important. Sham acupuncture is often used to control for expectancy, doctor–patient interaction, and procedural attention, but it is not always physiologically inert. Superficial needling and non-acupoint stimulation can activate cutaneous and somatosensory afferents, and systematic reviews have questioned whether sham acupuncture should be considered equivalent to an inactive placebo (30). In our study, sham acupuncture plus rehabilitation improved clinical scores but did not differ significantly from conventional rehabilitation, whereas manual acupuncture produced larger improvements than both. This finding may suggest that appropriate acupoint selection and adequate needling, including sufficient insertion depth, manual manipulation, and elicitation of de qi, contributed collectively to the observed therapeutic effect. This interpretation is consistent with sham-controlled evidence in stroke and stroke-related complications, which highlights the importance of these technical components and rigorous sham-controlled trial design in acupuncture research (31).

### 4.2 Interpretation of the integrated six acupoint protocol

One possible explanation for the effect of the six acupoint prescription is that it delivered repeated, spatially distributed somatosensory input from cranial, cervical, upper limb, and lower limb sites. GV20 and GV14 represented cranial and cervical stimulation sites, LI11 and PC6 represented upper limb sites, and ST36 and SP6 represented lower limb sites. Stimulation at these anatomically distinct sites may recruit partially different afferent pathways that converge at spinal and supraspinal levels, potentially providing complementary influences on corticospinal excitability, sensorimotor integration, and task related cortical recruitment (10, 11). Manual acupuncture is a physical stimulation intervention in which point selection, needle depth, manipulation style, stimulation duration, and treatment frequency may influence therapeutic effects (32). The elicitation of de qi is also relevant because de qi-related sensations are mediated by somatosensory nerve fibers and have been associated with cortical network modulation in neuroimaging studies (33). Because the six acupoints were evaluated only as an integrated prescription, the present trial cannot determine the contribution of individual acupoints or establish whether their combined effect was additive or synergistic. The proposed convergence of peripheral afferent, spinal, corticospinal, and sensorimotor network processes should therefore be regarded as a mechanistic hypothesis.

### 4.3 sEMG findings

The sEMG findings showed treatment-related differences in absolute RMS amplitudes during the recorded tasks. The sEMG has been increasingly used in upper-limb stroke rehabilitation to quantify muscle activation, co-contraction, spasticity-related responses, and recovery patterns (34). In the present study, voluntary-contraction RMS amplitudes increased more in the acupuncture group for the biceps brachii and triceps brachii than in the sham acupuncture and conventional rehabilitation groups. These proximal muscles contribute to elbow flexion and extension, and proximal upper-limb recovery often precedes more refined wrist and hand recovery after stroke (35). During passive stretch, significant group-by-time interactions were observed only for the triceps brachii and wrist flexor RMS. The direction of change was not uniform across muscles. Conventional rehabilitation produced a larger increase in stretch-evoked triceps brachii RMS than acupuncture, whereas the acupuncture group showed relative preservation in wrist flexor RMS while the conventional rehabilitation and sham acupuncture groups showed decreases. These findings do not support a simple conclusion that acupuncture globally reduced or increased muscle tone (36). Instead, they suggest muscle-specific modulation of stretch-responsive neuromuscular activity. This interpretation is consistent with previous work showing that stretch-evoked EMG after stroke is velocity-dependent, can reflect the neural component of spasticity, and may not correspond closely to clinical scales such as the Modified Ashworth Scale (37). Therefore, in the present study, the passive-stretch results should be regarded as exploratory evidence of treatment-related changes in reflex-mediated or stretch-responsive muscle activation rather than as direct proof of an antispastic effect.

### 4.4 fNIRS activation and connectivity findings

The fNIRS results provide exploratory evidence that the clinical improvements were accompanied by changes in cortical hemodynamic responses. fNIRS is well suited to stroke rehabilitation research because it can monitor task-related cortical hemodynamic responses and resting-state FC during clinically feasible motor paradigms. In our study, task-related HbO activation increased significantly in the ipsilesional PFC and ipsilesional M1 in the acupuncture group, but not in the sham acupuncture or conventional rehabilitation groups. The increased activation of the ipsilesional M1 is consistent with the central role of the lesioned motor cortex in post-stroke motor recovery. The M1 is a key cortical region for voluntary motor output, and greater recruitment of the ipsilesional motor cortex has been regarded as an important marker of adaptive motor network reorganization after stroke (38). Similarly, Chau et al. reported that acupuncture at LI4 and LI11 in patients with chronic stroke was associated with improved upper-limb motor function and more extensive and stronger activation of the lesioned sensorimotor cortex during a hand-grip task (39). Yuan et al. also found that acupuncture treatment elicited stronger activation in the ipsilesional M1 (40). Together, these findings suggest that greater ipsilesional M1 activation may represent an exploratory marker of task-related recruitment within the lesioned hemisphere. The PFC is involved in higher-order motor regulation, including attention allocation, movement planning, performance monitoring, and strategy adjustment. Recent fNIRS evidence in upper-extremity motor control tasks has shown that PFC HbO responses increase with task difficulty, indicating that the PFC contributes to the additional control resources required for more demanding upper-limb movements (41). In stroke populations, Wang et al. further demonstrated that prefrontal hemodynamic changes and PFC lateralization imbalance were associated with limb motor dysfunction, supporting the relevance of PFC activity as a neural marker of post-stroke motor status (42). Moreover, fNIRS studies of upper-limb rehabilitation have shown that task-related cortical responses across motor and prefrontal regions are associated with upper-limb performance and functional status in patients with stroke (43). Therefore, the increased ipsilesional PFC HbO response in the acupuncture group may reflect greater engagement of cognitive–motor control processes during paretic upper-limb task performance.

The resting-state connectivity findings help refine this exploratory interpretation. After treatment, FC in the acupuncture group was lower than that in both control groups for the interhemispheric ipsilesional PFC–contralesional PFC and ipsilesional PFC–contralesional PM connections, as well as for the contralesional PFC–contralesional PM connection. In contrast, ipsilesional M1–ipsilesional S1 FC increased in the acupuncture group. Previous fNIRS studies have shown that resting-state functional connectivity can characterize post-stroke upper-limb recovery, and that PFC–motor network connectivity is related to motor impairment severity and FMA-UE performance (44). However, increased PFC-PM coupling after stroke does not necessarily indicate more efficient recovery; it may also reflect compensatory top-down control, greater attentional demand, or increased motor-planning effort when sensorimotor execution remains inefficient. In this context, the combination of stronger task-evoked ipsilesional PFC and M1 activation with lower resting-state PFC–PFC and PFC–PM connectivity is compatible with a more focused pattern of task-related recruitment and less diffuse prefrontal involvement in the acupuncture group. This interpretation is consistent with broader stroke network models showing that motor recovery depends on dynamic interactions between ipsilesional and contralesional systems, and that excessive bilateral or contralesional recruitment is often associated with poorer or less complete recovery, whereas better recovery is commonly accompanied by a shift toward ipsilesional motor-system engagement (7). The increase in ipsilesional M1–S1 connectivity represents a complementary within-hemisphere neurophysiological finding. M1 and S1 form a reciprocal sensorimotor loop in which somatosensory feedback contributes to motor output, motor learning, and adaptive movement control (45). Stroke can disrupt this sensorimotor integration, and impairment of sensory feedback processing may contribute to inefficient voluntary movement. Therefore, the greater increase in ipsilesional M1–S1 connectivity in the acupuncture group may be consistent with altered coupling between somatosensory and motor cortical processes on the lesioned side. Taken together, this pattern provides an exploratory neurophysiological correlate of the clinical findings and generates a hypothesis that more focused ipsilesional sensorimotor organization may contribute to recovery.

### 4.5 Safety

Overall, no major safety concerns were identified. Only three mild needling-related bleeding events occurred, and all resolved with routine local management. No serious adverse events, infections, fainting, stuck needles, broken needles, recurrent stroke, clinically significant deterioration, or withdrawals due to adverse events were reported. This safety profile is consistent with broader acupuncture safety literature, which generally finds that minor adverse events such as bleeding, hematoma, and transient discomfort are more common than serious events, whereas serious acupuncture-related adverse events are rare when treatment is delivered by trained practitioners using appropriate procedures (46).

### 4.6 Limitations

Several points should be considered when interpreting the findings. The trial was conducted at a single center with a modest sample, and the intervention period was 4 weeks; longer follow-up is needed to determine the durability of treatment effects and whether continued acupuncture yields additional gains. The number of sEMG and fNIRS comparisons further supports interpreting the neurophysiological findings as exploratory, despite the use of multiplicity correction. Three participants underwent endpoint assessment before the scheduled week-4 visit because of early discharge, which may have introduced minor variation in assessment timing. Participants were within 3 months after ischemic stroke, so the findings are most directly applicable to early and subacute recovery rather than chronic stroke. Masking was also incomplete. The credibility of the sham acupuncture procedure and treatment expectancy were not formally assessed, and participants in the conventional rehabilitation group could not be blinded to the absence of needling, which may have introduced performance and expectation bias in comparisons involving this group. In addition, sEMG-derived RMS amplitudes were analyzed without normalization. Residual between-session variation due to electrode repositioning and skin–electrode conditions therefore cannot be excluded. Finally, fNIRS and sEMG provide valuable objective biomarkers, but they do not fully capture lesion anatomy, corticospinal tract integrity, or fine hand kinematics, and the two systems did not share a hardware trigger or common time base, so temporally aligned analysis of the two could not be performed. Future multicenter trials should include longer follow-up, stratification by lesion location and baseline impairment severity, and integrated multimodal biomarkers to identify patients most likely to benefit.

## 5 Conclusion

A 4-week standardized manual acupuncture protocol combined with conventional rehabilitation improved upper-limb motor function and activities of daily living in patients with ischemic stroke more than sham acupuncture or conventional rehabilitation alone. These clinical improvements were accompanied by larger increases in biceps brachii and triceps brachii RMS amplitudes during voluntary contraction and by exploratory fNIRS changes in ipsilesional PFC and M1 activation and resting-state functional connectivity. These neurophysiological findings represent treatment-related correlates and support further investigation of peripheral neuromuscular and cortical processes as potential contributors to recovery. The intervention was well tolerated, with only mild transient needling-related bleeding and no serious adverse events.

## Data Availability

The original contributions presented in the study are included in the article/Supplementary material, further inquiries can be directed to the corresponding author.
