# Supplementary material for: Clinical and neurophysiological effects of manual acupuncture for upper-limb motor dysfunction after ischemic stroke: a three-arm randomized controlled trial
Source: Front Neurol. 2026 Aug 25;17:1886740. doi: 10.3389/fneur.2026.1886740 (PMC13549818; doi:10.3389/fneur.2026.1886740)
Supplement: Supplementary file 1 [file Table_1.DOCX]

**Supplementary Table S1.** CONSORT 2025 Checklist

| **Section / Topic** | **No** | **CONSORT 2025 checklist item description** | **Reported on page no.** |
| --- | --- | --- | --- |
| **Title and abstract** | | |  |
| Title and structured abstract | 1a | Identification as a randomised trial | 1 |
|  | 1b | Structured summary of the trial design, methods, results, and conclusions | 1-2 |
| **Open science** | | |  |
| Trial registration | 2 | Name of trial registry, identifying number (with URL) and date of registration | 2, 6, 29 |
| Protocol and statistical analysis plan | 3 | Where the trial protocol and statistical analysis plan can be accessed | 6 |
| Data sharing | 4 | Where and how the individual de-identified participant data (including data dictionary), statistical code and any other materials can be accessed | 30 |
| Funding and conflicts of interest | 5a | Sources of funding and other support (e.g., supply of drugs), and role of funders in the design, conduct, analysis and reporting of the trial | 30 |
|  | 5b | Financial and other conflicts of interest of the manuscript authors | 30 |
| **Introduction** | | |  |
| Background and rationale | 6 | Scientific background and rationale | 2-5 |
| Objectives | 7 | Specific objectives related to benefits and harms | 5, 9 |
| **Methods** | | |  |
| Patient and public involvement | 8 | Details of patient or public involvement in the design, conduct and reporting of the trial | 6 |
| Trial design | 9 | Description of trial design including type of trial (e.g., parallel group, crossover), allocation ratio, and framework (e.g., superiority, equivalence, non-inferiority, exploratory) | 5-6 |
| Changes to trial protocol | 10 | Important changes to the trial after it commenced including any outcomes or analyses that were not prespecified, with reason | 6 |
| Trial setting | 11 | Settings (e.g., community, hospital) and locations (e.g., countries, sites) where the trial was conducted | 6 |
| Eligibility criteria | 12a | Eligibility criteria for participants | 6 |
|  | 12b | If applicable, eligibility criteria for sites and for individuals delivering the interventions (e.g., surgeons, physiotherapists) | 6, 8 |
| Intervention and comparator | 13 | Intervention and comparator with sufficient details to allow replication. If relevant, where additional materials describing the intervention and comparator (e.g., intervention manual) can be accessed | 8-9 |
| Outcomes | 14 | Pre-specified primary and secondary outcomes, including the specific measurement variable (e.g., systolic blood pressure), analysis metric (e.g., change from baseline, final value, time to event), method of aggregation (e.g., median, proportion), and time point for each outcome | 9-13 |
| Harms | 15 | How harms were defined and assessed (e.g., systematically, non-systematically) | 12-13 |
| Sample size | 16a | How sample size was determined, including all assumptions supporting the sample size calculation | 6-7 |
|  | 16b | Explanation of any interim analyses and stopping guidelines | 6-7 |
| Randomisation: |  |  |  |
| Sequence generation | 17a | Who generated the random allocation sequence and the method used | 7 |
|  | 17b | Type of randomisation and details of any restriction (e.g., stratification, blocking and block size) | 7 |
| Allocation concealment mechanism | 18 | Mechanism used to implement the random allocation sequence (e.g., central computer/telephone; sequentially numbered, opaque, sealed containers), describing any steps to conceal the sequence until interventions were assigned | 7 |
| Implementation | 19 | Whether the personnel who enrolled and those who assigned participants to the interventions had access to the random allocation sequence | 7 |
| Blinding | 20a | Who was blinded after assignment to interventions (e.g., participants, care providers, outcome assessors, data analysts) | 7 |
|  | 20b | If blinded, how blinding was achieved and description of the similarity of interventions | 7-9 |
| Statistical methods | 21a | Statistical methods used to compare groups for primary and secondary outcomes, including harms | 13 |
|  | 21b | Definition of who is included in each analysis (e.g., all randomised participants), and in which group | 13-14 |
|  | 21c | How missing data were handled in the analysis | 14 |
|  | 21d | Methods for any additional analyses (e.g., subgroup and sensitivity analyses), distinguishing prespecified from post-hoc | 13 |
| **Results** | | |  |
| Participant flow, including flow diagram | 22a | For each group, the numbers of participants who were randomly assigned, received intended intervention, and were analysed for the primary outcome | 14-15 |
|  | 22b | For each group, losses and exclusions after randomisation, together with reasons | 13-15 |
| Recruitment | 23a | Dates defining the periods of recruitment and follow-up for outcomes of benefits and harms | 6, 8, 14 |
|  | 23b | If relevant, why the trial ended or was stopped | N/A (completed) |
| Intervention and comparator delivery | 24a | Intervention and comparator as they were actually administered (e.g., where appropriate, who delivered the intervention/comparator, how participants adhered, whether they were delivered as intended [fidelity]) | 8, 14 |
|  | 24b | Concomitant care received during the trial for each group | 8 |
| Baseline data | 25 | A table showing baseline demographic and clinical characteristics for each group | 15 |
| Numbers analysed,  outcomes and estimation | 26 | For each primary and secondary outcome, by group:   - the number of participants included in the analysis - the number of participants with available data at the outcome time point - result for each group, and the estimated effect size and its precision (such as 95% confidence interval) - for binary outcomes, presentation of both absolute and relative effect size | 14-21 |
| Harms | 27 | All harms or unintended events in each group | 22-23 |
| Ancillary analyses | 28 | Any other analyses performed, including subgroup and sensitivity analyses, distinguishing pre-specified from post-hoc | 13-21 |
| **Discussion** | | |  |
| Interpretation | 29 | Interpretation consistent with results, balancing benefits and harms, and considering other relevant evidence | 23-29 |
| Limitations | 30 | Trial limitations, addressing sources of potential bias, imprecision, generalisability, and, if relevant, multiplicity of analyses | 28-29 |

| **Supplementary Table S2.** sEMG-derived RMS amplitudes during passive stretch | | | | | | | |
| --- | --- | --- | --- | --- | --- | --- | --- |
| Outcome | Time point | Conventional rehabilitation group | Sham acupuncture group | Acupuncture group | Between-group P | Interaction Wald χ² | Interaction P |
| Biceps brachii RMS, μV | Baseline | 15.22(11.60, 19.07) | 11.88(8.24, 20.47) | 17.39(8.56, 26.89) | 0.244 | 1.49 | 0.475 |
|  | Post-treatment | 16.84(14.16, 27.46) | 12.23(6.17, 21.74) | 17.86(5.88, 31.05) | 0.183 |  |  |
|  | Change from baseline (95% CI) | 3.54(0.44, 6.64) | 0.38(-3.67, 4.42) | -0.17(-6.85, 6.51) | 0.475 |  |  |
| Triceps brachii RMS, μV | Baseline | 3.04(2.08, 6.54) | 4.86(2.83, 8.44) | 6.83(2.23, 10.38) | 0.414 | 8.33 | 0.016 |
|  | Post-treatment | 12.39(4.83, 22.74) | 11.15(4.21, 18.76) | 7.60(1.56, 16.60) | 0.110 |  |  |
|  | Change from baseline (95% CI) | 12.17(6.68, 17.66) | 8.32(3.94, 12.70) | 2.70(-1.98, 7.39) | 0.016 |  |  |
| Wrist flexor RMS, μV | Baseline | 11.21(5.23, 20.70) | 7.76(5.31, 14.43) | 11.53(6.80, 17.54) | 0.280 | 19.75 | <0.001 |
|  | Post-treatment | 6.21(4.04, 8.14) | 5.41(1.98, 8.46) | 12.09(6.36, 17.34) | <0.001 |  |  |
|  | Change from baseline (95% CI) | -7.44(-11.10, -3.79) | -4.98(-7.91, -2.05) | 2.37(-1.18, 5.93) | <0.001 |  |  |
| Wrist extensor RMS, μV | Baseline | 7.10(1.70, 10.81) | 6.58(3.91, 11.46) | 5.49(2.06, 11.22) | 0.864 | 2.45 | 0.294 |
|  | Post-treatment | 10.95(8.24, 13.85) | 10.75(6.71, 13.64) | 7.88(2.41, 12.71) | 0.498 |  |  |
|  | Change from baseline (95% CI) | 3.40(0.76, 6.03) | 1.99(-0.31, 4.29) | 0.33(-3.27, 3.93) | 0.294 |  |  |
| Baseline and post-treatment values are presented as mean ± SD or median (IQR), as appropriate; estimated changes from baseline are presented with 95% CIs. sEMG, surface electromyography; RMS, root mean square; CI, confidence interval. | | | | | | | |

| **Supplementary Table S3.** sEMG-derived RMS amplitudes during voluntary contraction | | | | | | | |
| --- | --- | --- | --- | --- | --- | --- | --- |
| Outcome | Time point | Conventional rehabilitation group | Sham acupuncture group | Acupuncture group | Between-group P | Interaction Wald χ² | Interaction P |
| Biceps brachii RMS, μV | Baseline | 71.54±36.76 | 59.70±42.74 | 66.60±30.99 | 0.554 | 10.33 | 0.006 |
|  | Post-treatment | 79.91±44.02 | 70.21±32.18 | 105.69±57.54 | 0.004 |  |  |
|  | Change from baseline (95% CI) | 8.37(-7.40, 24.13) | 10.51(-4.55, 25.57) | 39.09(22.63, 55.55) | 0.006 |  |  |
| Triceps brachii RMS, μV | Baseline | 30.65(11.65, 49.63) | 39.66(23.76, 67.78) | 35.84(22.28, 52.42) | 0.552 | 29.94 | <0.001 |
|  | Post-treatment | 45.07(10.75, 78.94) | 50.48(20.48, 96.86) | 88.23(51.94, 113.48) | <0.001 |  |  |
|  | Change from baseline (95% CI) | 8.31(-4.80, 21.42) | 9.24(-3.14, 21.62) | 57.64(38.37, 76.91) | <0.001 |  |  |
| Wrist flexor RMS, μV | Baseline | 19.89(7.91, 31.27) | 27.65(8.33, 45.00) | 19.84(2.40, 52.43) | 0.168 | 4.92 | 0.086 |
|  | Post-treatment | 43.38(13.62, 59.31) | 36.78(17.19, 63.70) | 68.44(36.30, 77.53) | 0.003 |  |  |
|  | Change from baseline (95% CI) | 15.69(7.67, 23.71) | 9.65(0.04, 19.26) | 25.17(12.16, 38.19) | 0.086 |  |  |
| Wrist extensor RMS, μV | Baseline | 19.11(5.86, 37.46) | 18.24(6.10, 31.81) | 11.95(3.84, 29.65) | 0.844 | 0.40 | 0.817 |
|  | Post-treatment | 28.89(3.66, 66.39) | 30.80(18.75, 51.89) | 37.63(20.10, 59.91) | 0.714 |  |  |
|  | Change from baseline (95% CI) | 20.02(5.43, 34.60) | 17.77(10.37, 25.17) | 22.20(15.04, 29.36) | 0.817 |  |  |
| Baseline and post-treatment values are presented as mean ± SD or median (IQR), as appropriate; estimated changes from baseline are presented with 95% CIs. sEMG, surface electromyography; RMS, root mean square; CI, confidence interval. | | | | | | | |

| **Supplementary Table S4.** Task-related fNIRS cortical activation by ROI, ΔHbO (10⁻² mmol/L·mm) | | | | | | | | |
| --- | --- | --- | --- | --- | --- | --- | --- | --- |
| ROI | Time point | Conventional rehabilitation group | Sham acupuncture group | Acupuncture group | Between-group P | Interaction Wald χ² | Interaction P | FDR-adjusted interaction P value |
| l-PFC | Baseline | 4.02(1.21, 6.68) | 5.53(-0.91, 8.70) | 3.34(-0.35, 5.30) | 0.753 | 40.56 | <0.001 | <0.001 |
|  | Post-treatment | 2.14(0.14, 5.14) | 2.91(-0.47, 7.91) | 8.87(6.01, 11.04) | <0.001 |  |  |  |
|  | Change from baseline (95% CI) | -1.22(-3.05, 0.60) | -0.63(-2.61, 1.35) | 6.58(4.36, 8.80) | <0.001 |  |  |  |
| r-PFC | Baseline | 5.87(0.39, 11.44) | 6.22(-1.97, 10.37) | 1.74(-1.64, 7.87) | 0.641 | 1.93 | 0.380 | 0.622 |
|  | Post-treatment | 2.52(-0.24, 7.41) | 4.97(-1.06, 8.26) | 2.15(-1.20, 5.89) | 0.389 |  |  |  |
|  | Change from baseline (95% CI) | -1.56(-4.77, 1.66) | 1.05(-1.94, 4.04) | -0.85(-3.18, 1.47) | 0.380 |  |  |  |
| l-M1 | Baseline | 0.87(-2.25, 4.46) | 1.87(-0.23, 5.64) | 1.89(0.09, 4.63) | 0.539 | 9.07 | 0.011 | 0.043 |
|  | Post-treatment | 1.71(0.40, 3.13) | 3.32(-1.05, 7.76) | 7.75(3.86, 10.25) | <0.001 |  |  |  |
|  | Change from baseline (95% CI) | 1.31(-0.02, 2.64) | 1.62(-0.66, 3.89) | 4.93(2.76, 7.09) | 0.011 |  |  |  |
| r-M1 | Baseline | 3.62(1.00, 7.20) | 2.09(-1.79, 5.32) | 2.31(-2.15, 4.06) | 0.440 | 2.44 | 0.296 | 0.622 |
|  | Post-treatment | 4.74(1.59, 6.98) | 1.81(-4.24, 6.24) | 0.17(-2.30, 4.16) | 0.027 |  |  |  |
|  | Change from baseline (95% CI) | 1.20(-0.36, 2.75) | -0.21(-2.34, 1.93) | -1.17(-4.03, 1.70) | 0.296 |  |  |  |
| l-PM | Baseline | 1.28±2.77 | 0.78±3.55 | 2.27±4.10 | 0.443 | 1.23 | 0.541 | 0.622 |
|  | Post-treatment | 1.85±6.61 | 2.83±4.34 | 3.28±4.77 | 0.467 |  |  |  |
|  | Change from baseline (95% CI) | 0.57(-1.61, 2.75) | 2.04(0.60, 3.48) | 1.01(-1.29, 3.32) | 0.541 |  |  |  |
| r-PM | Baseline | 2.29(0.44, 4.88) | 2.03(-2.35, 4.47) | 2.38(0.05, 4.36) | 0.894 | 1.59 | 0.451 | 0.622 |
|  | Post-treatment | 1.31(-0.76, 3.69) | 1.62(-0.63, 5.10) | 0.85(-0.23, 3.85) | 0.778 |  |  |  |
|  | Change from baseline (95% CI) | -0.50(-1.50, 0.51) | 0.26(-1.19, 1.71) | -1.00(-2.88, 0.88) | 0.451 |  |  |  |
| l-S1 | Baseline | 3.34(0.92, 6.64) | 5.80(2.37, 7.94) | 4.04(-3.62, 7.92) | 0.270 | 0.95 | 0.622 | 0.622 |
|  | Post-treatment | 6.36(-0.95, 10.01) | 7.47(4.47, 9.46) | 5.90(3.11, 9.74) | 0.762 |  |  |  |
|  | Change from baseline (95% CI) | 2.68(-0.77, 6.14) | 2.49(0.34, 4.64) | 4.30(1.04, 7.56) | 0.622 |  |  |  |
| r-S1 | Baseline | 1.89(-1.22, 7.64) | 1.41(-4.49, 9.36) | 2.21(-2.46, 6.59) | 0.947 | 1.02 | 0.601 | 0.622 |
|  | Post-treatment | 1.39(-0.76, 3.82) | 4.09(-1.74, 8.92) | 2.49(-1.27, 5.64) | 0.530 |  |  |  |
|  | Change from baseline (95% CI) | -1.80(-4.78, 1.18) | 0.40(-4.06, 4.86) | 0.52(-3.35, 4.40) | 0.601 |  |  |  |
| Baseline and post-treatment values are presented as mean ± SD or median (IQR), as appropriate; estimated changes from baseline are presented with 95% CIs. fNIRS, functional near-infrared spectroscopy; ROI, regions of interest; PFC, prefrontal cortex; M1, primary motor cortex; PM, premotor cortex; S1, primary somatosensory cortex; FDR, false discovery rate; CI, confidence interval; l-/r-, ipsilesional/contralesional after lesion-side recoding. | | | | | | | | |

| **Supplementary Table S5.** Resting-state fNIRS functional connectivity between ROIs, Fisher z ×100 | | | | | | | | |
| --- | --- | --- | --- | --- | --- | --- | --- | --- |
| ROI | Time point | Conventional rehabilitation group | Sham acupuncture group | Acupuncture group | Between-group P | Interaction Wald χ² | Interaction P | FDR-adjusted interaction P value |
| l-PFC-r-PFC | Baseline | 55.81(15.60, 84.28) | 67.53(30.32, 95.99) | 82.49(54.70, 115.74) | 0.006 | 37.67 | <0.001 | <0.001 |
|  | Post-treatment | 84.27(66.59, 102.16) | 84.91(68.83, 89.32) | 43.06(29.62, 59.25) | <0.001 |  |  |  |
|  | Change from baseline (95% CI) | 33.59(14.73, 52.45) | 21.09(5.13, 37.05) | -41.49(-64.19, -18.80) | <0.001 |  |  |  |
| l-PFC-l-M1 | Baseline | 19.11±22.36 | 29.52±29.92 | 43.42±38.43 | 0.029 | 1.92 | 0.382 | 0.487 |
|  | Post-treatment | 49.07±27.45 | 66.14±37.95 | 63.89±47.70 | 0.130 |  |  |  |
|  | Change from baseline (95% CI) | 29.96(16.31, 43.62) | 36.62(16.18, 57.05) | 20.47(3.33, 37.60) | 0.382 |  |  |  |
| l-PFC-r-M1 | Baseline | 36.72±41.79 | 25.33±23.57 | 46.35±59.03 | 0.233 | 3.13 | 0.209 | 0.383 |
|  | Post-treatment | 42.39±59.05 | 56.23±50.09 | 49.26±38.23 | 0.533 |  |  |  |
|  | Change from baseline (95% CI) | 5.67(-26.26, 37.59) | 30.90(7.66, 54.14) | 2.92(-23.58, 29.41) | 0.209 |  |  |  |
| l-PFC-l-PM | Baseline | 31.43(11.66, 60.98) | 30.71(0.62, 46.60) | 42.27(19.93, 73.62) | 0.072 | 4.25 | 0.120 | 0.383 |
|  | Post-treatment | 43.31(26.85, 61.63) | 56.63(40.24, 80.12) | 52.97(26.05, 80.11) | 0.403 |  |  |  |
|  | Change from baseline (95% CI) | 7.50(-20.58, 35.58) | 32.30(9.68, 54.92) | 2.36(-19.90, 24.61) | 0.120 |  |  |  |
| l-PFC-r-PM | Baseline | 38.15±35.91 | 22.20±27.48 | 33.29±34.06 | 0.212 | 20.59 | <0.001 | <0.001 |
|  | Post-treatment | 78.75±52.08 | 64.10±28.18 | 22.96±28.22 | <0.001 |  |  |  |
|  | Change from baseline (95% CI) | 40.60(11.61, 69.60) | 41.90(23.98, 59.81) | -10.33(-25.03, 4.37) | <0.001 |  |  |  |
| l-PFC-l-S1 | Baseline | 34.66(1.75, 77.65) | 58.83(4.50, 102.32) | 50.29(6.92, 83.71) | 0.327 | 4.10 | 0.129 | 0.383 |
|  | Post-treatment | 80.04(55.49, 91.09) | 71.49(57.50, 84.94) | 63.44(46.37, 89.12) | 0.395 |  |  |  |
|  | Change from baseline (95% CI) | 43.48(19.32, 67.65) | 15.45(-11.93, 42.83) | 14.86(-4.50, 34.23) | 0.129 |  |  |  |
| l-PFC-r-S1 | Baseline | 64.66±53.09 | 34.31±23.76 | 56.53±37.57 | 0.044 | 3.53 | 0.171 | 0.383 |
|  | Post-treatment | 79.66±62.93 | 61.16±41.28 | 50.37±57.75 | 0.063 |  |  |  |
|  | Change from baseline (95% CI) | 15.00(-18.00, 48.00) | 26.85(5.36, 48.33) | -6.17(-33.30, 20.97) | 0.171 |  |  |  |
| r-PFC-l-M1 | Baseline | 44.40±26.26 | 36.64±33.38 | 53.11±58.40 | 0.222 | 2.20 | 0.333 | 0.474 |
|  | Post-treatment | 62.56±31.62 | 51.02±19.21 | 52.67±35.78 | 0.422 |  |  |  |
|  | Change from baseline (95% CI) | 18.16(1.93, 34.40) | 14.38(0.29, 28.48) | -0.44(-26.67, 25.79) | 0.333 |  |  |  |
| r-PFC-r-M1 | Baseline | 29.30(12.73, 58.18) | 26.06(13.27, 45.63) | 51.14(-0.67, 107.23) | 0.145 | 1.38 | 0.503 | 0.563 |
|  | Post-treatment | 35.09(5.88, 75.44) | 43.00(23.55, 70.82) | 44.05(23.59, 65.04) | 0.904 |  |  |  |
|  | Change from baseline (95% CI) | -1.95(-29.34, 25.45) | 10.44(-6.47, 27.34) | -10.07(-43.70, 23.57) | 0.503 |  |  |  |
| r-PFC-l-PM | Baseline | 49.68(-2.06, 99.28) | 26.50(4.93, 52.41) | 49.79(12.39, 88.81) | 0.045 | 2.81 | 0.245 | 0.383 |
|  | Post-treatment | 62.46(41.75, 90.78) | 40.67(27.53, 59.69) | 34.99(14.26, 56.13) | 0.123 |  |  |  |
|  | Change from baseline (95% CI) | 7.51(-32.09, 47.11) | 16.79(-1.25, 34.84) | -11.88(-35.05, 11.29) | 0.245 |  |  |  |
| r-PFC-r-PM | Baseline | 26.12±29.09 | 31.70±26.92 | 39.81±45.00 | 0.261 | 23.93 | <0.001 | <0.001 |
|  | Post-treatment | 56.53±31.63 | 52.51±25.99 | 19.90±29.31 | <0.001 |  |  |  |
|  | Change from baseline (95% CI) | 30.41(14.11, 46.71) | 20.81(7.46, 34.16) | -19.91(-38.27, -1.54) | <0.001 |  |  |  |
| r-PFC-l-S1 | Baseline | 63.37(31.36, 92.62) | 54.32(31.52, 72.47) | 59.08(4.35, 86.84) | 0.554 | 1.94 | 0.379 | 0.487 |
|  | Post-treatment | 46.49(31.40, 67.21) | 53.68(40.51, 68.26) | 56.26(44.29, 78.20) | 0.609 |  |  |  |
|  | Change from baseline (95% CI) | -12.04(-33.86, 9.78) | -4.20(-23.15, 14.75) | 8.97(-21.47, 39.41) | 0.379 |  |  |  |
| r-PFC-r-S1 | Baseline | 48.07±42.48 | 36.80±31.77 | 54.12±76.03 | 0.310 | 2.16 | 0.339 | 0.474 |
|  | Post-treatment | 36.42±25.11 | 46.99±28.04 | 50.44±38.69 | 0.446 |  |  |  |
|  | Change from baseline (95% CI) | -11.66(-30.21, 6.90) | 10.19(-6.20, 26.58) | -3.69(-33.12, 25.75) | 0.339 |  |  |  |
| l-M1-r-M1 | Baseline | 49.53±40.68 | 52.19±40.33 | 83.72±80.74 | 0.046 | 5.09 | 0.078 | 0.314 |
|  | Post-treatment | 105.96±77.78 | 90.50±55.79 | 91.90±38.10 | 0.536 |  |  |  |
|  | Change from baseline (95% CI) | 56.43(21.36, 91.51) | 38.30(13.73, 62.88) | 8.17(-26.80, 43.15) | 0.078 |  |  |  |
| l-M1-l-PM | Baseline | 48.08±56.85 | 54.99±50.23 | 72.17±58.67 | 0.180 | 3.85 | 0.146 | 0.383 |
|  | Post-treatment | 107.74±57.22 | 94.80±41.42 | 96.42±38.06 | 0.575 |  |  |  |
|  | Change from baseline (95% CI) | 59.66(30.17, 89.14) | 39.81(14.79, 64.84) | 24.25(-1.21, 49.70) | 0.146 |  |  |  |
| l-M1-r-PM | Baseline | 51.64±35.26 | 42.11±39.28 | 59.70±70.45 | 0.348 | 2.80 | 0.246 | 0.383 |
|  | Post-treatment | 83.83±43.12 | 75.82±46.37 | 68.31±32.58 | 0.441 |  |  |  |
|  | Change from baseline (95% CI) | 32.19(9.96, 54.43) | 33.72(8.54, 58.89) | 8.61(-18.30, 35.52) | 0.246 |  |  |  |
| l-M1-l-S1 | Baseline | 54.94(38.21, 74.68) | 52.26(22.25, 79.25) | 89.43(-0.72, 129.17) | 0.667 | 12.13 | 0.002 | 0.016 |
|  | Post-treatment | 56.54(40.10, 82.49) | 90.78(44.89, 130.89) | 129.85(117.32, 175.04) | <0.001 |  |  |  |
|  | Change from baseline (95% CI) | 1.57(-17.55, 20.68) | 34.82(5.28, 64.37) | 74.14(33.98, 114.30) | 0.002 |  |  |  |
| l-M1-r-S1 | Baseline | 38.92(4.04, 81.83) | 37.34(1.27, 65.37) | 46.47(4.23, 136.31) | 0.260 | 1.46 | 0.483 | 0.563 |
|  | Post-treatment | 78.52(46.05, 126.23) | 67.92(40.14, 86.84) | 79.76(49.26, 108.69) | 0.732 |  |  |  |
|  | Change from baseline (95% CI) | 36.34(1.95, 70.74) | 29.52(6.63, 52.41) | 8.58(-38.50, 55.65) | 0.483 |  |  |  |
| r-M1-l-PM | Baseline | 43.03±48.53 | 57.42±40.46 | 79.28±62.82 | 0.014 | 5.18 | 0.075 | 0.314 |
|  | Post-treatment | 62.94±52.02 | 70.26±38.10 | 63.21±38.66 | 0.802 |  |  |  |
|  | Change from baseline (95% CI) | 19.91(-9.83, 49.65) | 12.84(-10.12, 35.79) | -16.07(-36.71, 4.58) | 0.075 |  |  |  |
| r-M1-r-PM | Baseline | 41.89(20.93, 69.47) | 62.66(22.38, 90.68) | 68.93(35.52, 113.83) | 0.038 | 3.05 | 0.218 | 0.383 |
|  | Post-treatment | 75.83(49.30, 107.88) | 77.29(49.79, 117.36) | 77.03(52.60, 101.31) | 0.908 |  |  |  |
|  | Change from baseline (95% CI) | 26.99(5.00, 48.98) | 21.64(-4.46, 47.75) | -2.46(-33.29, 28.37) | 0.218 |  |  |  |
| r-M1-l-S1 | Baseline | 38.39±42.51 | 43.11±50.01 | 63.95±78.06 | 0.131 | 0.75 | 0.689 | 0.742 |
|  | Post-treatment | 69.50±42.12 | 70.66±34.98 | 79.54±49.50 | 0.718 |  |  |  |
|  | Change from baseline (95% CI) | 31.11(6.20, 56.01) | 27.55(4.39, 50.71) | 15.59(-18.56, 49.73) | 0.689 |  |  |  |
| r-M1-r-S1 | Baseline | 62.79±64.88 | 52.24±55.34 | 82.38±84.56 | 0.200 | 0.63 | 0.731 | 0.758 |
|  | Post-treatment | 70.95±50.05 | 78.42±59.48 | 105.01±69.83 | 0.110 |  |  |  |
|  | Change from baseline (95% CI) | 8.15(-26.62, 42.92) | 26.18(-4.14, 56.51) | 22.64(-21.45, 66.73) | 0.731 |  |  |  |
| l-PM-r-PM | Baseline | 63.31(29.90, 104.05) | 47.64(17.48, 75.76) | 71.56(28.43, 112.82) | 0.082 | 3.42 | 0.180 | 0.383 |
|  | Post-treatment | 82.91(68.76, 110.16) | 66.74(45.15, 99.54) | 64.86(47.31, 80.77) | 0.220 |  |  |  |
|  | Change from baseline (95% CI) | 20.05(-6.96, 47.05) | 21.28(-3.62, 46.19) | -6.91(-34.41, 20.59) | 0.180 |  |  |  |
| l-PM-l-S1 | Baseline | 25.94(7.30, 69.63) | 35.62(20.96, 75.75) | 42.47(-18.72, 89.63) | 0.593 | 0.12 | 0.941 | 0.941 |
|  | Post-treatment | 65.54(44.81, 88.94) | 81.08(46.05, 104.91) | 82.85(62.47, 103.31) | 0.530 |  |  |  |
|  | Change from baseline (95% CI) | 31.88(8.52, 55.24) | 29.99(7.68, 52.31) | 35.48(10.25, 60.71) | 0.941 |  |  |  |
| l-PM-r-S1 | Baseline | 14.92(0.55, 30.24) | 18.67(-7.03, 41.99) | 50.32(17.39, 103.24) | 0.005 | 5.96 | 0.051 | 0.285 |
|  | Post-treatment | 69.83(51.09, 100.00) | 64.13(46.87, 85.02) | 66.71(46.43, 81.19) | 0.753 |  |  |  |
|  | Change from baseline (95% CI) | 52.55(31.58, 73.52) | 40.10(20.59, 59.61) | 15.65(-11.37, 42.66) | 0.051 |  |  |  |
| r-PM-l-S1 | Baseline | 44.26±53.67 | 37.62±49.69 | 55.60±55.10 | 0.372 | 1.67 | 0.434 | 0.529 |
|  | Post-treatment | 75.89±48.50 | 50.93±44.22 | 65.57±43.28 | 0.152 |  |  |  |
|  | Change from baseline (95% CI) | 31.63(6.32, 56.93) | 13.31(-14.02, 40.63) | 9.97(-17.37, 37.31) | 0.434 |  |  |  |
| r-PM-r-S1 | Baseline | 35.99±47.71 | 33.23±41.18 | 57.64±90.58 | 0.171 | 3.38 | 0.184 | 0.383 |
|  | Post-treatment | 68.22±35.99 | 71.33±41.54 | 62.62±49.50 | 0.825 |  |  |  |
|  | Change from baseline (95% CI) | 32.24(12.18, 52.29) | 38.11(17.98, 58.23) | 4.98(-35.16, 45.12) | 0.184 |  |  |  |
| l-S1-r-S1 | Baseline | 80.01±59.81 | 52.11±48.66 | 63.41±96.68 | 0.250 | 2.94 | 0.230 | 0.383 |
|  | Post-treatment | 62.27±47.70 | 62.00±44.55 | 81.66±72.10 | 0.409 |  |  |  |
|  | Change from baseline (95% CI) | -17.75(-47.00, 11.51) | 9.89(-15.03, 34.82) | 18.25(-22.80, 59.30) | 0.230 |  |  |  |
| Baseline and post-treatment values are presented as mean ± SD or median (IQR), as appropriate; estimated changes from baseline are presented with 95% CIs. fNIRS, functional near-infrared spectroscopy; ROI, regions of interest; PFC, prefrontal cortex; M1, primary motor cortex; PM, premotor cortex; S1, primary somatosensory cortex; FDR, false discovery rate; CI, confidence interval; l-/r-, ipsilesional/contralesional after lesion-side recoding. | | | | | | | | |

| **Supplementary Table S6.** Post hoc pairwise comparisons of change scores | | | | | |
| --- | --- | --- | --- | --- | --- |
| Outcome | Effect type | Statistic | Conventional rehabilitation vs sham acupuncture | Conventional rehabilitation vs acupuncture | Sham acupuncture vs acupuncture |
| FMA-UE | Difference in change, ΔΔ | ΔΔ(95% CI) | -1.07(-5.09, 2.95) | -9.64(-13.66, -5.62) | -8.57(-12.59, -4.55) |
|  |  | Z | -0.52 | -4.70 | -4.18 |
|  |  | P value | 0.602 | <0.001 | <0.001 |
|  |  | Bonferroni-adjusted P value | >0.999 | <0.001 | <0.001 |
| MBI | Difference in change, ΔΔ | ΔΔ(95% CI) | 3.96(-1.84, 9.77) | -7.32(-13.13, -1.52) | -11.29(-17.09, -5.48) |
|  |  | Z | 1.34 | -2.47 | -3.81 |
|  |  | P value | 0.181 | 0.013 | <0.001 |
|  |  | Bonferroni-adjusted P value | 0.542 | 0.040 | <0.001 |
| Stretch-evoked triceps brachii RMS | Difference in change, ΔΔ | ΔΔ(95% CI) | 3.85(-2.61, 10.32) | 9.47(3.00, 15.93) | 5.61(-0.85, 12.08) |
|  |  | Z | 1.17 | 2.87 | 1.70 |
|  |  | P value | 0.243 | 0.004 | 0.089 |
|  |  | Bonferroni-adjusted P value | 0.729 | 0.012 | 0.266 |
| Stretch-evoked wrist flexor RMS | Difference in change, ΔΔ | ΔΔ(95% CI) | -2.47(-6.97, 2.04) | -9.81(-14.32, -5.31) | -7.35(-11.85, -2.84) |
|  |  | Z | -1.07 | -4.27 | -3.20 |
|  |  | P value | 0.283 | <0.001 | 0.001 |
|  |  | Bonferroni-adjusted P value | 0.849 | <0.001 | 0.004 |
| Biceps brachii RMS during voluntary contraction | Difference in change, ΔΔ | ΔΔ(95% CI) | -2.14(-23.06, 18.78) | -30.72(-51.64, -9.80) | -28.58(-49.50, -7.66) |
|  |  | Z | -0.20 | -2.88 | -2.68 |
|  |  | P value | 0.841 | 0.004 | 0.007 |
|  |  | Bonferroni-adjusted P value | >0.999 | 0.012 | 0.022 |
| Triceps brachii RMS during voluntary contraction | Difference in change, ΔΔ | ΔΔ(95% CI) | -0.93(-21.14, 19.28) | -49.33(-69.54, -29.12) | -48.40(-68.61, -28.19) |
|  |  | Z | -0.09 | -4.78 | -4.69 |
|  |  | P value | 0.928 | <0.001 | <0.001 |
|  |  | Bonferroni-adjusted P value | >0.999 | <0.001 | <0.001 |
| l-PFC | Difference in change, ΔΔ | ΔΔ(95% CI) | -0.59(-3.27, 2.08) | -7.80(-10.48, -5.13) | -7.21(-9.89, -4.54) |
|  |  | Z | -0.43 | -5.72 | -5.29 |
|  |  | P value | 0.664 | <0.001 | <0.001 |
|  |  | Bonferroni-adjusted P value | >0.999 | <0.001 | <0.001 |
| l-M1 | Difference in change, ΔΔ | ΔΔ(95% CI) | -0.31(-2.92, 2.30) | -3.62(-6.23, -1.01) | -3.31(-5.92, -0.70) |
|  |  | Z | -0.23 | -2.72 | -2.48 |
|  |  | P value | 0.816 | 0.007 | 0.013 |
|  |  | Bonferroni-adjusted P value | >0.999 | 0.020 | 0.039 |
| l-PFC-r-PFC | Difference in change, ΔΔ | ΔΔ(95% CI) | 12.50(-13.19, 38.20) | 75.09(49.39, 100.78) | 62.58(36.89, 88.28) |
|  |  | Z | 0.95 | 5.73 | 4.77 |
|  |  | P value | 0.340 | <0.001 | <0.001 |
|  |  | Bonferroni-adjusted P value | >0.999 | <0.001 | <0.001 |
| l-PFC-r-PM | Difference in change, ΔΔ | ΔΔ(95% CI) | -1.29(-27.03, 24.44) | 50.93(25.20, 76.66) | 52.22(26.49, 77.96) |
|  |  | Z | -0.10 | 3.88 | 3.98 |
|  |  | P value | 0.922 | <0.001 | <0.001 |
|  |  | Bonferroni-adjusted P value | >0.999 | <0.001 | <0.001 |
| r-PFC-r-PM | Difference in change, ΔΔ | ΔΔ(95% CI) | 9.60(-11.81, 31.01) | 50.32(28.91, 71.73) | 40.72(19.31, 62.12) |
|  |  | Z | 0.88 | 4.61 | 3.73 |
|  |  | P value | 0.379 | <0.001 | <0.001 |
|  |  | Bonferroni-adjusted P value | >0.999 | <0.001 | <0.001 |
| l-M1-l-S1 | Difference in change, ΔΔ | ΔΔ(95% CI) | -33.26(-74.15, 7.64) | -72.58(-113.47, -31.68) | -39.32(-80.21, 1.58) |
|  |  | Z | -1.59 | -3.48 | -1.88 |
|  |  | P value | 0.111 | <0.001 | 0.060 |
|  |  | Bonferroni-adjusted P value | 0.333 | 0.002 | 0.179 |
| ΔΔ, between-group difference in change from baseline; CI, confidence interval; FMA-UE, Fugl-Meyer assessment of the upper extremity; MBI, modified barthel index; RMS, root mean square; PFC, prefrontal cortex; M1, primary motor cortex; PM, premotor cortex; S1, primary somatosensory cortex; l-/r-, ipsilesional/contralesional after lesion-side recoding. | | | | | |
